# Effects of Electrical Stimulation of Raphe Magnus on Locomotion and Selected Cognitive Abilities in Rats

**DOI:** 10.3390/ijms27104215

**Published:** 2026-05-09

**Authors:** Kacper Ptaszek, Grażyna Jerzemowska, Karolina Plucińska, Artur H. Świergiel, Magdalena A. Zabielska-Kaczorowska

**Affiliations:** 1 Department of Physiology, Faculty of Medicine, Medical University of Gdansk, Debinki 1 Str, 80-210 Gdansk, Poland; kacper.ptaszek@gumed.edu.pl; 2 Department of Animal and Human Physiology, Faculty of Biology, University of Gdansk, Wita Stwosza 59 Str, 80-308 Gdansk, Poland; grazyna.jerzemowska@ug.edu.pl (G.J.); karolina.plucinska@ug.edu.pl (K.P.); artur.swiergiel@pan.pl (A.H.Ś.); 3 Polish Academy of Sciences, Defilad Sq 1, 00-901 Warsaw, Poland

**Keywords:** raphe magnus, serotonin, electrical stimulation, rat behavior, immunohistochemistry

## Abstract

Serotonin (5–HT) in the brain is involved in the regulation of various emotional states and behaviors. Most serotonergic neurons are located in the raphe nuclei. The raphe magnus (RMg) is one of the raphe nuclei and belongs to the caudal raphe complex. The primary goal of our research was to examine the effects of chronic, repeated electrical stimulation of the RMg on rats’ motility over a period of 15 days. During the research, 35 rats were used; 21 rats underwent electrical stimulation of the RMg (RMg-ST), while 14 rats were included in the control group (RMg-Sham). In addition, we aimed to evaluate the effects of electrical stimulation in the RMg-ST group as well as the naïve procedure in the RMg-Sham group on anxiety-related behaviors and spatial memory on selected days 30 min after the end of stimulation. We found that rats in the RMg-ST group were characterized by considerably higher locomotor activity than animals in the RMg-Sham group over a 15-day stimulation period. Stimulated animals were less anxious during the elevated plus maze on the 4th and 5th days of stimulation and demonstrated improved memory performance during the Morris water maze conducted between the 9th and 12th days of stimulation in comparison to the control animals. Furthermore, in both behavioral tests, rats’ motility when subjected to the RMg electrical stimulation was much higher than in control rats. On the last day of the 15-day stimulation period, rats were sacrificed, and their brains were collected. Brain immunofluorescent analysis revealed an increase in the number of 5–HT-positive cells in the RMg-ST group and altered activity of c-Fos-positive cells in selected brain structures connected with locomotion (secondary motor cortex), anxiety (arcuate nucleus of the hypothalamus), and spatial memory (dentate gyrus) after stimulation in comparison to the results in the RMg-Sham group. These findings suggest that locomotion may be strictly dependent on the RMg neuronal projections, and electrical stimulation of the structure influences cognitive behaviors.

## 1. Introduction

Serotonin (5–HT) is an endogenous amine present in the tissues of vertebrates, invertebrates, and even plants. In mammals, 5–HT is located peripherally, mainly in the blood platelets and intestinal cells [1]. Of particular interest, due to its complexity and broad influence on organisms, is the role of brain 5–HT. The serotonergic system is among the earliest-developing neurotransmitter systems [2] and becomes one of the most widespread, linking numerous brain regions [3]. Despite 5–HT being the most extensively distributed neurotransmitter in the brain, central 5–HT constitutes less than 5% of the total body content [4]. It is well known that central serotonergic neurons project to the cortex and limbic structures [5]. The serotonergic system is considered to be a regulator of numerous physiological functions, including sensory information processing [6], cognitive regulation, and regulating emotions [7].

In the brain, 5–HT cell bodies are organized into groups of neurons known as B groups, which together form the raphe system [4]. In terms of anatomy, the raphe nuclei are situated in the middle of the reticular formation, resembling a ridge of cells in the middle and most medial part of the brainstem. Neurons within these systems project both rostrally and caudally. The frontally projecting neurons originate in the most rostrally localized cell bodies [8]—the median raphe nucleus (B8) and the dorsal raphe nucleus (B6)—and are more diffusely localized around the lemniscus medialis. The caudally projecting neurons originate from cell bodies in B1–B3 [3].

The raphe magnus (RMg), also known as B3, is made up of about 30,000 neurons and is the largest 5–HT-producing nucleus in the caudal group of raphe nuclei. Only about a quarter of the neurons (15–20%) within this nucleus are serotonergic [9]. Serotonergic neurons in the RMg discharge in a characteristic slow (1–2 spikes/s) and steady discharge pattern [10]. The caudal raphe complex, including RMg, mainly projects to the spinal cord and cerebellum [4]. The RMg is considered a key relay in the descending pain control pathway to the dorsal horn [11]. In addition, electrical stimulation of this area reduces pain and stops spinal and trigeminal nociceptive neurons from responding [12]. The structure has also been described as being involved in the compensatory responses to hypoxia [13]. Additionally, RMg plays a role in thermoregulation by controlling the blood flow to the skin and the metabolism of brown adipose tissue [14]. It also plays a significant role in regulating neural activity associated with respiration [15]. In particular, the roles of serotonergic neurons in the RMg in the sleep–wake cycle and in somatomotor, autonomic, and behavioral responses to pain and thermal stimuli are also well understood [9].

The interaction between the serotonergic system and the RMg in regulating locomotion is complex. RMg is recognized for transmitting serotonergic projections to the spinal cord, influencing motoneurons, and initiating body movements. Conversely, serotonergic neurons located in the RMg are among the largest neurotransmitter systems in the central nervous system. Thus, the primary objective of the present research was to investigate the impact of chronic and repetitive electrical stimulation of RMg in rats on their locomotor activity. Since the RMg is one of the critical caudal structures of the serotonergic system, during post-mortem brain analyses, we performed double immunostaining of 5–HT-positive (5–HT+) cells and c-Fos-positive (c-Fos+) nuclei of the structure. c-Fos protein is a marker of neuronal activity in the brain. The serotonergic system has also been implicated in the modulation of mental or cognitive functions. Therefore, we decided to broaden our analysis of RMg electrical stimulation effects in rats by incorporating two tests on selected days 30 min after the end of stimulation: the elevated plus maze (EPM), which examines anxiety-related behaviors, and the Morris water maze (MWM), which examines spatial memory. These tests also allow for the assessment of the animals’ motility. Additionally, we expanded our research by analyzing the density of (c-Fos+) nuclei in brain structures associated with the behavioral measurements. We chose to examine the secondary motor cortex (M2), which is associated with locomotor activity; the arcuate nucleus (ARC) of the hypothalamus, linked to anxiety-related behaviors; and the dentate gyrus (DG), connected to spatial memory.

## 2. Results

### 2.1. Experimental Timeline

**Figure 1:**
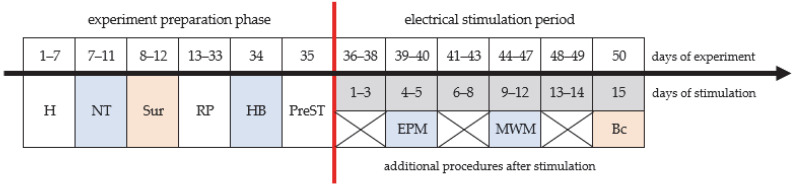
Schematic diagram of experimental timeline. Abbreviations used: H: handling; NT: novelty test; Sur: stereotactic surgery—electrode implantation into the RMg; RP: recovery period in home cage; HB: day of habituation to the stimulation room and maze; PreST: pre-stimulation; EPM: elevated plus maze; MWM: Morris water maze; Bc: euthanized and brain collected.

The entire experiment consisted of two stages: (1) an experimental preparation phase and (2) an electrical stimulation period (Figure 1). During the preparation phase, all rats (N = 35) were acclimated to human contact (handling). Rats’ locomotor activity in the new environment was also determined in a novelty test (NT). The mean value calculated for the entire group of 35 rats was equal to 4517 movements in 120 min. The whole group of 35 rats also underwent surgical implantation of an electrode in the RMg, followed by a 21-day recovery period. After recovery, the animals were habituated to the stimulation room and the stimulation maze (habituation day). The final procedure of the experimental preparation phase involved assessing the rats’ behavioral response to increasing electrical current intensity administered through a stimulating electrode placed in the RMg (pre-stimulation) directly into the structure. Only on the basis of the responses during pre-stimulation were the rats assigned to the stimulated (RMg-ST; n = 21 rats) or control (RMg-Sham; n = 14 rats) groups.

### 2.2. Rats’ Locomotor Response in the Novelty Test

The NT was conducted on the last day of handling. Efforts were made to ensure that the RMg-ST and RMg-Sham groups did not differ in terms of rats’ locomotor response to the new environment. The mean score for the animals assigned to the RMg-ST group (n = 21) was 1798.7 horizontal movements, whereas the control group (n = 14) had a mean equal to 1839.8 movements. The results of both groups met the assumptions of normal distribution (Lilliefors significance level below 0.05). A statistical analysis using the *t*-test showed that the two groups did not differ significantly (t_df=33_ = 0.195, *p* = 0.85).

### 2.3. Raphe Magnus Stimulation Effects on Locomotor Activity

The next day after pre-stimulation, the second phase of the experiment began—the electrical stimulation period. Electrical stimulation (or the naïve procedure in control rats) was carried out in the same room and maze as the NT. The stimulation procedure lasted 15 days and was repeated in the same way every day. The first session began at 8:00 a.m. and lasted 30 min for each rat, with subsequent sessions every 45 min until 1:15 p.m. (six sessions in total). A two-way ANOVA indicated that group assignment had a significant effect on locomotor activity during the stimulation (F_(1, 1)_ = 381.394, *p* < 0.001). The interaction between group assignment and individual days also significantly affected locomotor activity (F_(1, 14)_ = 6.594, *p* = 0.02). In contrast, the effect of stimulation day alone was not significant (F_(1, 14)_ = 1.229, *p* = 0.25).

**Figure 2:**
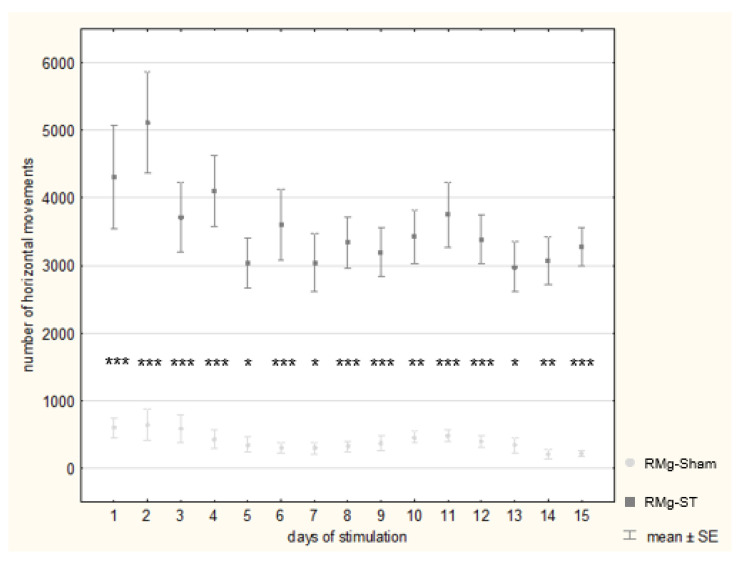
Comparison of locomotor activity in rats during electrical stimulation of the RMg (RMg-ST group) or the naïve procedure in the RMg-Sham group. The two-way ANOVA indicates a significant interaction between stimulation group and day (F_(14, 495)_ = 6.594, *p* = 0.02). Post hoc comparisons reveal highly significant differences between groups (* *p* < 0.05; ** *p* < 0.01; *** *p* < 0.001).

Each day, rats included in the RMg-ST group exhibited significantly higher locomotor activity compared to the RMg-Sham group results (Figure 2). The highest values were observed during the first days of the stimulation: on the 1st day, the mean activity was 4310.1 in the stimulated group and 593.1 in the control group, and on the 2nd day, it was 5114.1 and 644.9, respectively. Based on the Games–Howell post hoc analysis of the group × day effects, the differences between the groups on these days were statistically significant. Throughout the entire procedure, locomotor activity in the rats with the RMg electrical stimulation remained over three times higher than that of the control animals. Behavioral procedures in the tests followed stimulation; the EPM on the 4th to 5th days and the MWM on the 9th to 12ths day did not significantly affect locomotor activity during stimulation.

### 2.4. Raphe Magnus Stimulation Effects on Anxiety

The EPM assesses anxiety-related behavior in animals. To minimize the impact of stress induced by novel experimental conditions, the test was conducted over two consecutive days, the 4th and 5th days of the stimulation period, with the 1st day treated as an adaptation session and the 2nd as the actual test. Data from the two days were compared using a paired *t*-test (the distance in the maze, the duration of movement, the latency to the first movement, the time spent in the open arms and zone transition within the closed arms) or a Wilcoxon signed-rank test (the number of entries into the open arms). On both days, 30 min after the end of stimulation (rats from the RMg-ST group; n = 21) or the naïve procedure (rats from the RMg-Sham group; n = 14), the animals (N = 35) were transferred to the behavioral testing room and, following a 5 min adaptation period, the EPM was initiated. The first session began at 9:05 a.m. and lasted 5 min for each rat, with subsequent sessions every 45 min until 2:15 p.m. (six sessions in total).

**Figure 3:**
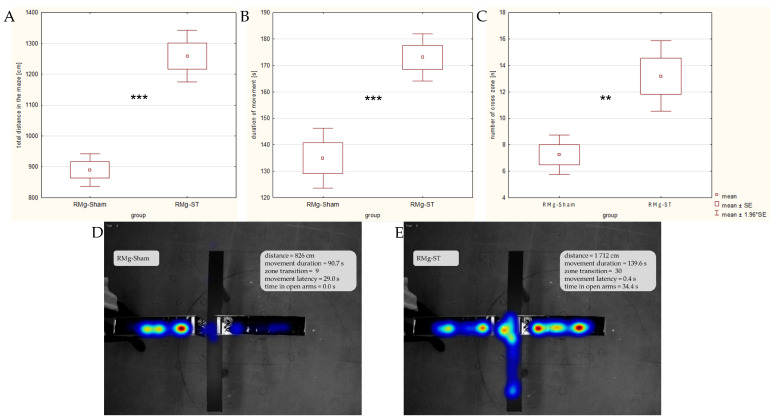
Comparison of rats’ motility: the total distance in the maze (**A**), the duration of movement (**B**) and zone transition (**C**) on the 2nd day of testing in the EPM. The heatmaps highlight the differences in motility between an exemplary individual from control (**D**) and stimulated (**E**) animal, as well as their exploration habits within the maze areas. Heatmap color explanations: the blue color shows the paths the rats followed where they spent more than 5 s; the green color shows the places where the rats spent more than 10 s; and the red color shows the places where they spent more than 20 s. The *t*-test indicates a significant difference between groups (*** *p* < 0.001) in the total distance and the duration of movement as well as (** *p* < 0.01) in the number of zone transitions.

The locomotor activity of rats subjected to the RMg electrical stimulation was significantly higher. The distance traveled in the maze (mean distance 1258.5 cm in the RMg-ST group and 889.7 cm in the RMg-Sham group; Figure 3A) differed significantly between groups (normal distribution confirmed; t_df=33_ = −6.495, *p* = 0.000). Duration of the movement (Figure 3B) was also significantly higher (normal distribution confirmed; t_df=33_ = −3.280, *p* = 0.001) in rats with the electrical stimulation of the RMg (mean time of 173.0 s in the RMg-ST group) compared with control animals (134.9 s).

Rats with the electrical stimulation of the RMg (mean 13.2 episodes in the RMg-ST group) also crossed between the arms (zone transition; Figure 3C) significantly more often (normal distribution confirmed; t_df=33_ = −3.321, *p* = 0.01) than rats with the naïve procedure (7.3 episodes). Similarly, rats with the electrical stimulation of the structure (mean 5.4 episodes in the RMg-ST group) entered the open arms significantly more frequently (distribution deviated from normality; Mann–Whitney U test: Z_U=36.0_ = −3.779, *p* = 0.001) than animals in the control group (2.6 episodes).

**Figure 4:**
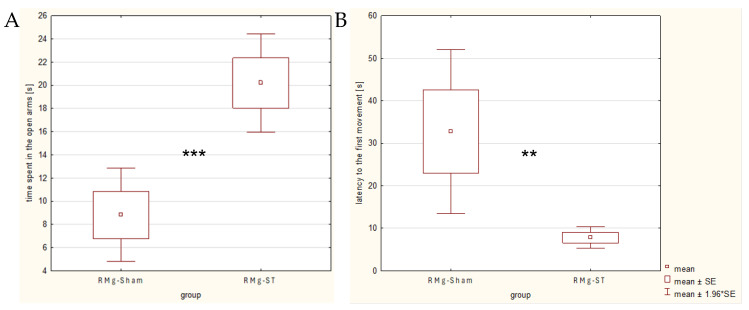
Comparison of the time spent in the open arms (**A**) and the latency to the first movement of rats (**B**) on the 2nd day of testing during the EPM. The *t*-test indicates a significant difference between groups (** *p* < 0.01; *** *p* < 0.01) in both parameters.

In addition, rats subjected to RMg electrical stimulation (mean time 20.2 s in the RMg-ST group) spent significantly more time in the open arms (normal distribution confirmed; t_df=33_ = −3.627, *p* = 0.001; Figure 4A) compared with rats with the naïve procedure (8.8 s). The latency to the first movement (Figure 4B) was significantly shorter (normal distribution confirmed; t_df=33_ = −3.073, *p* = 0.01) in the RMg-ST group (mean time 7.8 s) than in the RMg-Sham group (32.7 s).

Time spent in the open arms and the latency to the first movement are considered indicators of anxiety levels in rats. A two-way ANOVA indicated that motility (group × distance) may influence latency time (F_(1, 66)_ = 21.594, *p* = 0.00), whereas no such effect was observed for time spent in the open arms (group × distance; F_(1, 66)_ = 3.467, *p* = 0.00). These results indicate a significant anxiolytic effect of RMg electrical stimulation in the tested animals, especially in the case of their time spent in the open arms.

### 2.5. Raphe Magnus Stimulation Effects on Spatial Memory

The MWM assesses spatial and reference memory in animals. The MWM was conducted over four consecutive days on the 9th to 12th days of the stimulation period. Every four days, 30 min after the end of electrical stimulation (RMg-ST) or a naïve procedure (RMg-Sham), the animals were transferred to the behavioral testing room and, following a 5 min adaptation period, the MWM was initiated. The first session began at 9:05 a.m. and lasted 2 min for each rat, with subsequent sessions every 45 min until 2:15 p.m. (six sessions in total).

The 1st day of the MWM was treated as a training session. All animals (N = 35: n = 21 in the RMg-ST group and n = 14 in the RMg-Sham) successfully completed both trials scheduled for that day and were therefore included in the testing on the subsequent days. The mean latency to reach the platform during that day was equal to 33.2 s for the RMg-ST group, whereas for the control animals it was 37.6 s, with no significant difference between the groups (normal distribution confirmed; t_df=68_ = 1.235, *p* = 0.22). In contrast, the distance swum by the stimulated group (mean 762.9 cm) was significantly lower (normal distribution confirmed; t_df=68_ = 2.664, *p* = 0.05) compared with the control group (1010.6 cm).

**Figure 5:**
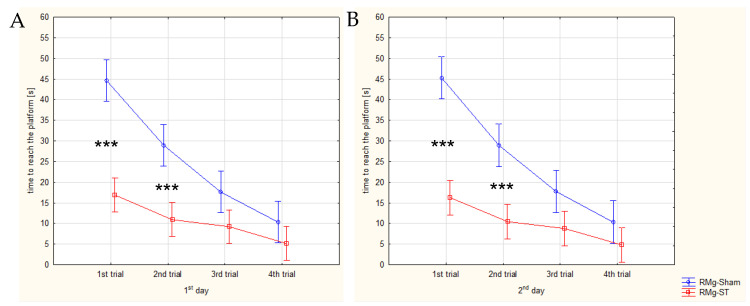
Comparison of the latency to reach the platform during the spatial memory test on the 1st day (**A**) and 2nd day (**B**) in the MWM. The two-way ANOVA indicates a significant interaction between stimulation group and day (F_(1, 264)_ = 8.391, *p* = 0.000). Post hoc comparisons reveal highly significant group × trial differences (*** *p* < 0.001).

On the 2nd and 3rd days of the MWM, spatial memory was assessed (1st and 2nd days of the spatial memory test; Figure 5). On these days, the platform was placed below the water surface; thus, the rat had to recall its location and locate it in the maze. Four trials were conducted each day. A two-way ANOVA was performed for the latency to reach the platform. A significant effect of group assignment was found (F_(1, 1)_ = 167.121, *p* = 0.000), as well as an effect of trial order (F_(1, 7)_ = 30.614, *p* = 0.000), and a significant interaction between group and trial (F_(7, 264)_ = 8.391, *p* = 0.000). The Games–Howell post hoc analysis confirmed significant differences in latency to reach the platform during trials 1 and 2 on both days between the RMg-ST and RMg-Sham groups.

The latencies to reach the platform were on average 16.9 s in the RMg-ST group compared with 44.7 s in the RMg-Sham group on the 1st day of the spatial memory test, and 16.3 s in the RMg-ST group compared with 45.3 s in the RMg-Sham group on the 2nd day of testing, during the 1st trial. For the rats with the electrical stimulation of the RMg, during the 2nd trial, the latencies were 10.9 s on the 1st day and 10.5 s on the 2nd day, whereas the control animals with the naïve procedure reached the platform in a mean time of 29.0 s on both the 1st and 2nd days.

Differences between individual trials within each day were also analyzed; however, no statistically significant differences were found between trial pairs within the same day. These results were additionally examined using paired-samples tests within each day and group (paired-samples *t*-test or Wilcoxon signed-rank test), but they were not included due to lower statistical power compared with the two-way ANOVA.

Faster identification and reaching of the platform in the maze indicate that electrical stimulation of the RMg had a positive effect on spatial memory in rats.

A statistical analysis based on a two-way ANOVA was also performed for the distance swum by the rats during the spatial memory test. Similarly, a significant effect of group assignment was found (F_(1, 1)_ = 120.894, *p* = 0.000), as well as a significant effect of trial (F_(1, 7)_ = 34.862, *p* = 0.000) and a significant group × trial interaction (F_(7, 264)_ = 5.664, *p* = 0.000). The Games–Howell post hoc analysis confirmed significant differences in the distance swum during the 1st and 2nd trials on both days between the RMg-ST and RMg-Sham groups. The statistically significant results confirm a markedly shorter distance swum by the rats with the electrical stimulation of the RMg during the spatial memory test in the MWM. The distance swum in the maze averaged 630.2 cm in the RMg-ST group compared with 1484.1 cm in the RMg-Sham group on the 1st day of the spatial memory test, and 618.3 cm in the RMg-ST group compared with 1401.8 cm in the RMg-Sham group on the 2nd day of testing, during the 1st trial. For the rats with the electrical stimulation of the RMg, during the 2nd trial, the distances were 297.4 cm on the 1st day and 280.6 cm on the 2nd day, whereas the control animals with the naïve procedure swam an average of 765.8 cm on the 1st day and 725.0 cm on the 2nd day. Differences between individual trials within each day were also analyzed; however, no statistically significant differences were found between trial pairs within the same day.

**Figure 6:**
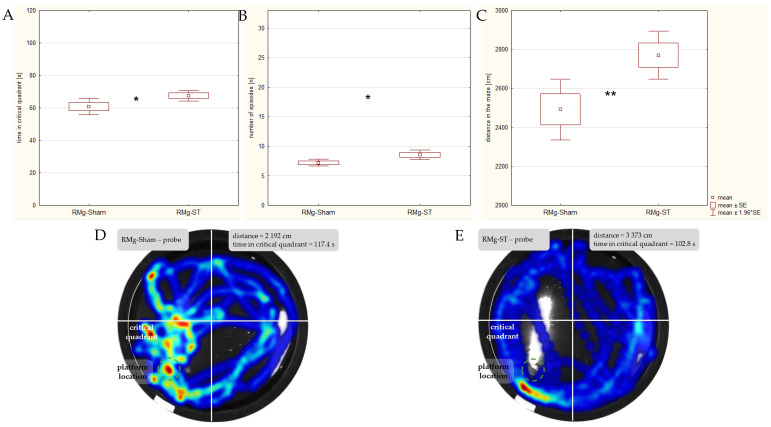
Comparison of parameters during the probe phase in the MWM: the time spent in the critical quadrant (**A**), entries into the critical quadrant (**B**), and the distance swum in the maze (**C**). The heatmaps highlight the differences in distance swum between an exemplary individual from the RMg-Sham (**D**) and RMg-ST (**E**) groups, as well as rats’ distinct patterns of spatial exploration within the maze. Heatmap color explanations: the blue color shows the paths the rats followed where they spent more than 5 s; the green color shows the places where the rats spent more than 10 s; and the red color shows the places where they spent more than 20 s. The *t*-test indicates a significant difference between groups (* *p* < 0.05) for the time spent in the critical quadrant and number of entries into the critical quadrant as well as (** *p* < 0.01) for the distance swum in the maze.

Reference memory was analyzed during the probe phase on the 4th day of the MWM (12th day of the stimulation period). On this day, the platform was removed from the maze, and the measurement lasted a full 2 min. Rats in the RMg-ST group spent a total of 67.5 s (Figure 6A) in the quadrant where was the platform previously (critical quadrant), and this value was significantly higher (normal distribution confirmed; t_df=33_ = 2.230, *p* = 0.03) compared with the RMg-Sham group, which spent 60.4 s in the critical quadrant. Rats with the electrical stimulation of the RMg also entered the critical quadrant (mean 8.6 entries; Figure 6B) significantly more frequently (normal distribution confirmed; t_df=33_ = 2.473, *p* = 0.02) compared with animals from the RMg-Sham group (7.2 entries). These findings suggest that electrical stimulation of the RMg also enhanced the animals’ reference memory.

The ability to swim freely in the MWM during the probe phase resulted in a significantly greater distance traveled by rats (Figure 6C) in the RMg-ST group (mean 2770.9 cm) compared with the RMg-Sham group (2492.2 cm) (normal distribution confirmed; t_df=33_ = 2.473, *p* = 0.02). In contrast, the total number of crossings between quadrants, which averaged 29.6 episodes in the RMg-ST group and 27.5 episodes in the RMg-Sham group, did not differ significantly (normal distribution confirmed; t_df=33_ = −1.250, *p* = 0.22).

### 2.6. Raphe Magnus Stimulation Effects on the Brain Structures

**Figure 7:**
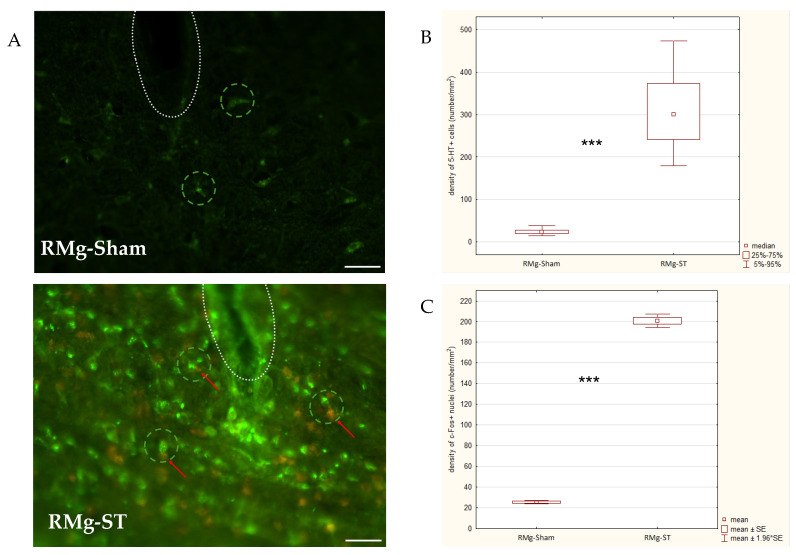
Digital images taken through a microscope (micrographs; (**A**)) of double staining with the density of 5–HT+ cells (green signal) and with the density of c-Fos+ nuclei (red signal) of the RMg in representative rats from the control (RMg-Sham) and stimulation (RMg-ST) groups. Comparison of the density of 5–HT+ cells (**B**) and the density of c-Fos+ nuclei (**C**) in the RMg following electrical stimulation of the RMg or the naïve procedure in control rats. Additional explanations for micrograph: the white dotted line indicates the trace of the stimulating electrode, the green dashed line indicates an example of the presence of 5–HT+ cells, and the red arrow shows an example of the presence of c-Fos+ nuclei; scale bar = 25 μm: white line in lower right corner of the micrographs. The Mann–Whitney U test indicates a significant difference between groups (*** *p* < 0.001) for 5–HT+ cells. The *t*-test indicates a significant difference between groups (*** *p* < 0.001) for c-Fos+ nuclei.

Quantitative immunofluorescence analyses (Figure 7A) revealed significantly higher densities of both 5–HT+ cells and c-Fos+ nuclei in the RMg-ST group compared with the RMg-Sham group. For the 5–HT+ cells (Figure 7B), the results of the RMg-ST group were 313.4 cells/mm^2^, a significantly increased density per mm^2^ (distribution deviated from normality; Z_U=0.0_ = −12.037, *p* = 0.000) relative to the RMg-Sham group results (24.9 cells/mm^2^). Similarly, for c-Fos+ nucleus density (Figure 7C), the RMg-ST group (200.8 nuclei/mm^2^) exhibited substantially higher activation marker expression (normal distribution confirmed; t_df=202_ = 42.448, *p* = 0.000) compared with the RMg-Sham group (25.4 nuclei/mm^2^).

Overall, these findings indicate that electrical stimulation of the RMg significantly increases both 5–HT+ cell density and neuronal activation (c-Fos expression) within the examined region (area frame equal to 49 591 µm^2^).

**Figure 8:**
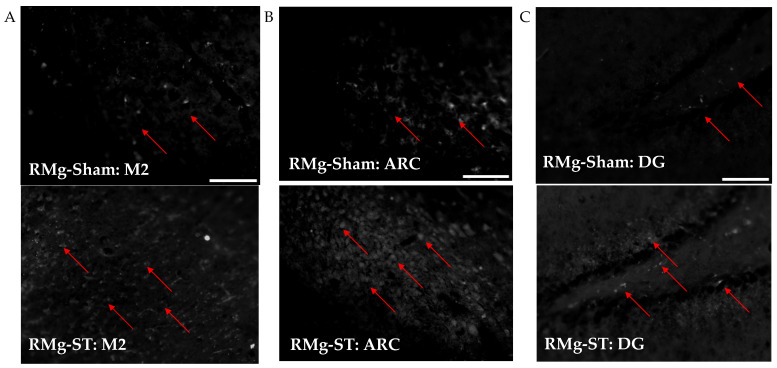
Micrographs of the density of c-Fos+ nuclei in M2 (**A**), ARC (**B**), and DG (**C**) in representative rats from the control (RMg-Sham) and stimulation (RMg-ST) groups. Additional explanations for micrograph: the red arrow shows an example of the presence of c-Fos+ nuclei; scale bar = 40 μm: white line in lower right corner of the images in the first row.

Quantitative immunofluorescence analysis of c-Fos+ cell density across three brain structures, M2 (Figure 8A), ARC (Figure 8B), and DG (Figure 8C), revealed marked and statistically significant increases in neuronal activation in the RMg-ST group compared with the RMg-Sham group.

Analyzed brain sections from the RMg-ST group exhibited a substantially higher density of c-Fos+ nuclei than those from the control group in the M2. The median value in the RMg-ST group equals 108, calculated for the 206 brain sections, whereas in the RMg-Sham group it was 11, calculated for the 136 brain sections. Similarly, c-Fos+ nucleus density in the ARC was significantly increased in the RMg-ST group (median of 133 calculated for the 101 brain sections) compared with the control group (median of 23 calculated for the 67 brain sections). An increase in activation was also observed in the DG, where the results of sections taken from the RMg-ST group were characterized by a substantially higher density of c-Fos+ nuclei (mean of 112.1 calculated for the 208 brain sections) relative to the control group (mean of 13.4 calculated for the 136 brain sections).

**Figure 9:**
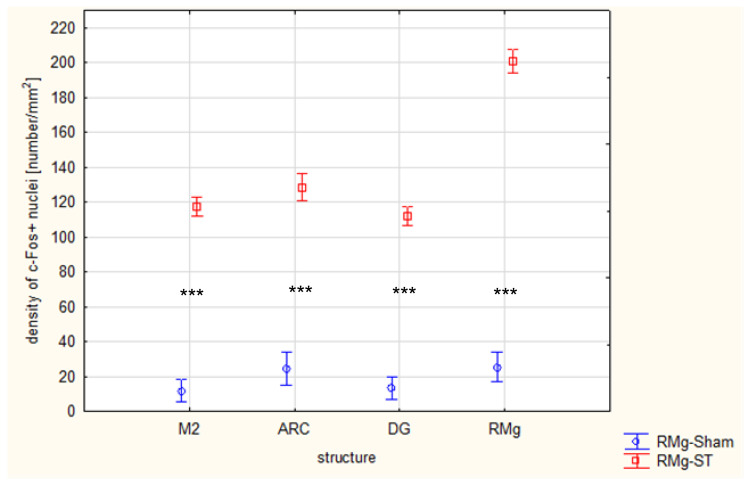
Comparison of the density of c-Fos+ nuclei in selected brain structures: M2, ARC, DG, and RMg in rats following RMg electrical stimulation (RMg-ST) or the naïve procedure in the control (RMg-Sham) group. The two-way ANOVA confirms a significant interaction between stimulation group and brain structure (F_(3, 1050)_ = 46.684, *p* = 0.000). Post hoc comparisons reveal highly significant group × structure differences (*** *p* < 0.001).

Across all examined structures—M2, ARC, and DG—electrical stimulation of the RMg resulted in a pronounced increase in neuronal activation, as indicated by significantly elevated c-Fos expression (Figure 9). The consistency of these effects across multiple brain regions highlights the widespread impact of the RMg electrical stimulation on neural activity. This result is additionally supported by a two-way ANOVA, in which the interaction effect between group and brain structure was statistically significant (F_(3, 1050)_ = 46.684, *p* = 0.000).

## 3. Discussion

Electrical stimulation is considered an appropriate method for studies focused on neuronal activity, gene and protein expression, neurotransmitter levels, neuroinflammation, neuroprotection, and neural or synaptic plasticity [16]. Asahina et al. indicated that RMg electrical stimulation in cats affects skin blood flow [14]. Single-cell firing analysis revealed that RMg afferent neurons are linked to 5–HT release at their terminals [17]. Although the caudal raphe nuclei mainly project to the spinal cord and cerebellum [4], electrophysiological studies from the 1990s have since challenged this view. Firstly, Lumb and Wolstencroft demonstrated rostral projections from the RMg to the hypothalamus in both rats and cats [18]. Secondly, studies by Carstens et al. confirmed neuronal projections from the RMg to both the hypothalamus and the lateral thalamus [19]. Furthermore, the RMg contains various cell types, including serotonergic neurons, which contribute to the structural and functional complexity of this region [10]. For instance, serotonergic neurons in the RMg are modulated by local GABAergic inputs [9]. Furthermore, the connection between the RMg and the hypothalamus in pain modulation may be regulated by arginine vasopressin [20]. Another important aspect concerns the activity of the serotonergic system, which depends on the type of serotonergic receptors involved. For instance, 5–HT_1A_ receptors in the RMg modulate hypoxia-induced hyperpnea [13] and increase ventilatory responses [21].

In the present study, we conducted chronic and repetitive electrical stimulation of the RMg throughout a 15-day period. Our literature review indicates that this study is the first to examine the effects of RMg electrical stimulation in freely moving rats. The primary behavioral effect observed during the electrical stimulation of the RMg was an increase in locomotor activity. It occurred approximately 3–5 s after each current stimulation episode and lasted up to 2 s after its end. The number of horizontal movements in the RMg-ST group was approximately three times higher than in the RMg-Sham animals with the naïve procedure throughout the 15-day stimulation period. Understanding the brain mechanisms of locomotion is crucial for neuroscience research. Previous findings suggested that cortical involvement during walking is unnecessary in rats, as this movement is an unconscious, rhythmic form of locomotion that is primarily controlled by spinal cord motor networks [22]. Recent studies have indicated that the extent of cortical involvement in walking control likely relies on the environmental conditions [23], especially those that do not depend solely on the various locomotion terrains (e.g., walking on an upslope vs. downslope) but may be the result of new or enriched environmental conditions. For instance, factors such as the presence of obstacles, varying surface textures, and social interactions can significantly influence how the brain engages in locomotion [24]. In the 1990s, Garcia-Rill et al. already suggested that the spinal cord and posterior midbrain structure could induce locomotion [25]. It is now well established that multiple brain structures contribute to the control of locomotion. We conducted an NT and analyzed the results to ensure that both the RMg-ST and RMg-Sham groups included either low- or high-activity rats in their response to the new environment. We know that this response affects many aspects, such as other rats’ behaviors [26] or brain structure organization [27]. Individual responses to a new environment also affect the structure and metabolic activity of 5–HT neurons [7]. These findings highlight the complex relationship between environmental factors and neural responses in the case of the locomotion. Understanding these dynamics could lead to a better understanding of locomotion control.

Rats are animals that like dark and confined spaces, which provide them with a sense of security. Additionally, these animals are characterized by an innate desire to explore new areas [28]. The EPM enables the determination of anxiety levels by utilizing the animal’s contradictory tendencies: exploring a new environment while being exposed to open space [29]. In our experiment, rats with RMg electrical stimulation responded quickly (approximately 10 s) after the EPM began. In contrast, control animals (without electrical stimulation) did not move for four times longer, accounting for almost 15% of the total time measured in the maze. A longer latency time can be treated as an indicator of a higher level of anxiety. Kiyokawa et al. also evidenced that the 5–HT system suppresses avoidance behavior while the dopaminergic system enhances the behavior of approaching novel objects in rats [30]. Another significantly different result is the time spent in the open arms. Rats, in general, explored the potentially dangerous open area less; nevertheless, the results of the animals with the electrical stimulation of the RMg were significantly higher. Behavioral data were recorded and analyzed using video software (EthoVision XT10), which enabled precise measurement of multiple parameters. One of them was the distance the rats covered in the maze. Animals with RMg electrical stimulation were significantly more active. The other advantage of the program was the ability to create heatmaps that showed the rats’ movement paths and locations within the maze. Animals from the control group with only the naïve procedure tended to remain in one place, mainly in the distal area of the closed arms. In contrast, rats with the electrical stimulation of the RMg actively explored the maze, even reaching the ends of the open arms. It is known that fear and anxiety are coordinated by the serotonergic regulation [31]. The B8 serotonergic projections to the hippocampus are crucial in conditioned fear [32]. Furthermore, these B8 projections involve both the prefrontal cortex and amygdala [33]. Nevertheless, the mechanism may not be straightforward, as dos Santos et al. have indicated that serotonergic neurons in the B8 regulate inhibitory avoidance but not escape behavior, suggesting that different serotonergic pathways may be involved in these distinct types of fear responses [34]. This highlights the complexity of the serotonergic system and its multifaceted role in fear processing. Future research should focus on elucidating the specific pathways and interactions involved to better understand how these mechanisms contribute to various fear-related behaviors.

To assess the functioning of both spatial and reference memory, the MWM was performed. Rats in the RMg-ST group found the platform faster, not only during the training stage (when the platform was visible) but also in the later days of the MWM (when the platform was under the water surface). On days 2 and 3 of the MWM, the animals had to remember the platform’s location and learn how to reach it. Faster platform finding time indicates better spatial memory [35], which was noted in our research in the RMg-ST group in comparison to the RMg-Sham. Additionally, in the probe phase, rats with the electrical stimulation of the RMg spent most of their time in the quadrant where the platform had been located on previous days. These rats more often also crossed zones, tried to locate the platform in that quadrant, and were in the particular location of the platform. These results may be related to an improvement in reference memory [36]. In general, cholinergic systems have been linked to cognitive processes, including attention, learning, and memory function [37]. However, other neurotransmitter systems, such as the serotonergic system, which may have only minor effects on cognitive function on their own, interact with cholinergic function, and their combined effects may have marked behavioral actions [38]. Fernandez et al. found in mice that both constitutive and acquired 5–HT deficiency impair memory, particularly in novel-object recognition and procedural learning. Furthermore, 5–HT deficiency also inhibited hippocampal synaptic plasticity [39]. López-Vázquez et al. indicated that enhanced hippocampal 5–HT activity influences working memory. 5–HT from the B8 desynchronizes theta activity in the hippocampus, thereby regulating its activity. Inactivation or a lesion of the B8 induced continuous and persistent theta activity in the hippocampus. However, these effects may be inhibited by medial septum 5–HT depletion, as it increases hippocampal high-frequency theta activity [40]. Wolff et al. indicated that spatial memory skills are age-dependent. In that process, 5–HT and 5–HT_1A_ receptors played a crucial role [41]. Conversely, studies analyzing 5–HT involvement in spatial memory suggest that the role of this system is more extensive than previously thought and requires further investigation [42].

The c-Fos protein, encoded by the proto-oncogene *c-fos*, belongs to the family of immediate early gene products involved in transcriptional regulation. Its expression may be induced to high levels by physiological stimuli and it is reported to be transiently expressed in neurons after synaptic stimulation. Immunostaining for c-Fos is a well-established marker of neuronal activity and functional pathways [43]. The brain slice immunohistochemical analysis clearly shows that rats with RMg electrical stimulation produced more c-Fos protein after the stimulation period compared to the control group with the naïve procedure. This effect was observed either near the electrode location (in the RMg) or in structures known to be involved in locomotion (M2) and cognitive functions (ARC and DG). Increased levels of c-Fos protein in the DG were also observed in rats after B6 activation [44]. Immunohistochemical c-Fos analysis also indicated that the projection from the limbic system (including structures such as the hippocampus and hypothalamus) via the subpallidal region to the mesencephalon (including raphe nuclei) is involved in the initiation and maintenance of locomotor activity [45]. This may also explain the higher c-Fos protein production in all of the analyzed structures in our research after the RMg electrical stimulation.

The importance of the motor cortex in regulating locomotion is well established [46]. Electrical stimulation of the RMg in our study significantly increased the number of c-Fos+ nuclei in the M2 compared to the control group with the naïve procedure. The density of c-Fos+ nuclei in the M2 is the second highest among the analyzed structures. Locomotion induction and regulation involve other structures, including the hippocampus [5], the lateral septum [47], the lateral pallidum [48], and the nucleus accumbens [49]. Locomotion, as a critical ability in animals, is present at birth and is primarily controlled by the brainstem [50]. The serotonergic system plays a crucial role in regulating locomotion. 5–HT depletion in rat pups induced impairments in the locomotion profile in both newborns and adults [51]. Serotonergic innervation, especially to the hippocampus, modulated locomotor activity [5]. Crucial activity, for instance, is carried out by afferents from the midbrain raphe region, which appear to suppress locomotor initiation [52]. Additionally, while forebrain-targeting 5–HT neurons (especially those starting in B6) decrease their activity during locomotion, and subpopulations of spinal-projecting neurons (especially those starting in the RMg) increase their activity in a context-dependent manner [53]. Furthermore, serotonergic neurons projecting to the hippocampus, but not to the striatum, modulated rat locomotor activity [54]. There is also more evidence of the participation of the rostral raphe nuclei in locomotor responses. For instance, B6 Deep Brain Stimulation (DBS), a more specific method than electrical stimulation, inhibited avoidance and escape reactions in rats [55]. Additionally, B6 DBS significantly enhanced hyperlocomotion and affected the polymorphic layer of the DG in the dorsal hippocampus. In contrast, B8 DBS had a greater effect on movement initiation and significantly altered serotonergic efferent innervations in the nucleus accumbens [44].

The behavioral results observed in the EPM in our research (especially lower latency and increased time spent in the open arms) suggest that RMg electrical stimulation has anxiolytic effects in rats. The immunofluorescence results also confirmed this. We observed that the density of c-Fos+ nuclei in the ARC is higher in rats after RMg electrical stimulation compared to control animals with the naïve procedure. It was even the highest response among the structures that we analyzed in terms of behaviors. B6 DBS induced anxiolytic as well as panicolytic-like effects in the EPM as well [56]. In turn, B8 DBS seems to participate in the modulation of conditioned anxiety but not unconditioned fear [57]. Electrical activation of the raphe nuclei increased c-Fos immunoreactivity in the serotonergic cells of the RMg (as observed in our research), the B6 [56], and the B8 [57]. These findings provide evidence for the essential role of the serotonergic system in anxiety responses [58]. It is well established that the regulation of anxiety within the serotonergic system involves two receptor types: 5–HT_1_ receptors, which are highly active in the paraventricular nucleus of the hypothalamus, and 5–HT_2_ receptors, which are present in the ARC [59]. Both hypothalamic structures transmit excitatory signals within the hypothalamic–pituitary–adrenal axis, thereby promoting the production of glucocorticoids. Moreover, they were associated with the regulation of anxiety levels [60]. The ARC is a brain structure that is closely linked to the regulation of anxiety [61]. On one hand, the ARC projects to the raphe nuclei, thereby modulating nociception [62]. On the other hand, acute stress increases neuropeptide Y mRNA levels within the ARC [63]. In the case of fear memory, serotonergic projections from the B8 to the ventral hippocampus, which activate the structure, are crucial. That process has been additionally regulated by the corticotropin-releasing factor type 2 receptor, which plays a significant role in modulating the stress response and influencing fear memory consolidation [64].

The behavioral results from the MWM in our research indicated that RMg stimulation improved spatial memory. We also observed a higher density of c-Fos+ nuclei in the DG, a structure associated with memory. 5–HT release in the hippocampus plays a role in normal memory function by inhibiting the activity of CA1 neurons. Furthermore, the 5–HT_1A_ receptor is one of the main inhibitory receptors expressed in the hippocampus, and a high-intensity 5–HT_1A_ transcript can be observed in the pyramidal neurons of the CA1 region [39]. Recent studies have implicated hippocampal neurogenesis in learning- and memory-related tasks, such as contextual discrimination and spatial navigation [65]. Spatial memory caused neuronal changes, especially in the DG [66]. While serotonergic projections play a role in hippocampus memory functions, they are primarily modulated by cholinergic signals [67] which suggests that the interaction between these neurotransmitter systems is crucial for optimal memory performance. Furthermore, reduced 5–HT levels in rat pups were found not to affect spatial memory [68].

The behaviors of the rats, either in the EPM or MWM, clearly demonstrated the influence of the electrical stimulation of the RMg, as shown in the following test results after the electrical stimulation (or the naïve procedure in control animals). The results showed a significant difference between animals subjected to electrical stimulation of the RMg and those in the control group with the naïve procedure. Immunohistochemical procedures also provided evidence that RMg electrical stimulation influenced the structures responsible for the analyzed behaviors. Rostral raphe nuclei (the B6 and the B8) sent innervations to the limbic system [69] (including the hypothalamus and hippocampus). From one point of view, RMg electrical stimulation may activate rostral raphe nuclei because of interconnections between them [70], which may cause the effects observed in our experiment.

We are aware of this study’s limitations. Due to the consistency of results across trials on the 1st and 2nd days of the spatial memory test of the MWM, it is necessary to extend this phase from two to three or more days. We tried to adjust the stimulation duration so that it was not taxing on the animals but still allowed for the addition of behavioral testing. We planned a minimum of two days between behavioral procedures to ensure that the results of one test did not influence the other or affect changes in the brain, particularly the density of c-Fos+ nuclei. Perhaps a better strategy would be to conduct independent stimulation and individual behavioral tests with a longer interval between stimulation and behavioral testing. Electrical stimulation is a nonspecific method for stimulating neurons. Despite most rats being stimulated with a low current (approximately 110 μA in 14 of 21 rats), the RMg, as well as areas adjacent to the electrode, remained activated. Stimulation parameters should also be optimized for the next study, especially since we relied on one behavior (increased locomotor activity) as a result of direct electrical stimulation of the RMg. Furthermore, serotonergic neuron cells can be observed only in the brainstem. Therefore, our analysis focused exclusively on the density of 5–HT+ cells within the RMg. We conducted immunohistochemical analysis of combined 5–HT+/c-Fos+ in various brain structures, and these results are included in the Supplementary Materials. Nevertheless, the immunofluorescent detection of 5–HT+ cells in the analyzed structures (M2, DG, and ARC) may indicate the presence of serotonin-containing axonal terminals (ascending originating in the raphe nuclei) or serotonergic receptors. Therefore, we did not analyze these results in detail. The other limitation is that we used only male rats in our research. There is evidence that gender influences the differential activity of the serotonergic system, particularly in situations related to stress [71].

Further research on RMg stimulation is warranted. Firstly, we analyzed the impact of RMg electrical stimulation strictly after the end of the stimulation period. Consequently, it is essential to assess the long-term effects of electrical stimulation, particularly on behavior. Secondly, we would like to examine the results of 5–HT detection in the analyzed brain structures using higher specificity antibodies. Lastly, the mechanism underlying the connections between the RMg and other raphe nuclei remains poorly understood. We believe that it should be analyzed. There is also a lack of information on how functionally diverse RMg is. The comprehension is limited to the more prominent raphe nuclei, specifically B6 and B8. [72].

## 4. Materials and Methods

### 4.1. Animals

Male Wistar rats (N = 35), aged 3.5 months (14 weeks) and weighing 240–275 g at the time of surgery, were used for the experiments. Upon arrival at our animal facility, the rats were separated into groups of five animals per home cage (60 cm × 38 cm × 20 cm; cage #IV; UNO Life Science Solutions, Zevenaar, The Netherlands) and allowed one week to acclimate to our facility. They were then inspected twice daily, at approximately 8:00 a.m. and 3:00 p.m., to check for signs of stress. At this time rats were housed under standard laboratory conditions with ad libitum access to a standard rat diet (Labofeed B standard, Morawski, Poland) and water. After the acclimatization period, the experiment preparation phase began (Figure 1). The experimental timeline as well as all procedures were approved by the local ethical committee in Gdansk, Poland (decision number 38/2013, obtained on 14 October 2013), for the care and use of laboratory animals. This research was also in accordance with the EU Directive 2010/63/EU.

The first procedure of the experiment was handling, performed twice daily, at 8:00 a.m. and 3:00 p.m., which involved the rats being removed from the cage, held by the researcher, and placed in the hands and arms of the researcher. This procedure lasted a week and was intended to reduce the animals’ anxiety resulting from contact with the researcher and subjecting them to further experimental procedures.

### 4.2. Novelty Test

On the last day of the handling procedure, at 3:00 p.m., the NT was conducted. It assessed locomotor activity of rats in response to a novel environment and helped to divide animals into high- and low-responders. This trait is associated with a range of physiological characteristics of the animals. We used the standard protocol for this test [26]. The following summarizes the most important aspects of the setup: the novel environment was a clear Plexiglas maze (43 cm × 43 cm × 20 cm) equipped with 15 photoelectric cells placed on the perpendicular axis of the maze (Opto Varimex Minor, Columbus, OH, USA). The rats were placed in the maze for 120 min (3:00–5:00 p.m.), during which their horizontal activity was automatically recorded. For each animal, the number of photocell counts cumulated over 120 min was used to index individual reactivity to the new environment. After completing the NT, the rats were placed back in their home cage. Due to the extended duration of the NT, only data obtained during the first 30 min were included in the analysis described in the results. Furthermore, due to the limited number of actometers, the NT was performed in 9 rats during the day, so the measurement period for the entire group lasted 4 days.

### 4.3. Stereotaxic Implantation of Stimulating Electrode into the Raphe Magnus

The day after the NT, the animals underwent surgery to implant an electrode into the RMg. Stereotactic surgery was performed according to the previously described protocol [73]. The rats were anesthetized with 1.5–2.5% isoflurane (airflow: 0.5 L per min) using an isoflurane pump (Bitmos OXY 6000, Bitmos GmbH, Düsseldorf, Germany), and analgetic butorphanol (2.0 mg/kg; Butomidor, Richter Pharma, Wels, Austria) was administered subcutaneously. For this experiment, the monopolar stimulation electrodes for RMg were handmade using stainless-steel wire with a diameter of 0.2 mm and insulated with epoxy varnish along the entire length, except for the flat cut end (type 008SW, Bilaney Consultants GmbH, Düsseldorf, Germany). The stereotaxic coordinates from RMg were as follows: −10.2 mm posterior to the bregma, 0.0 mm lateral to the midline, and −10.2 mm ventral to the skull surface [74]. A stainless-steel wire was soldered to a screw, which was attached to the skull, and together they served as the anode for the electrical stimulation. The implanted stimulation electrode, the anode, and four additional stainless-steel screws were anchored to the skull with dental acrylic. Rats received an antibiotic solution (penicillin procaine, Polfa, Warsaw, Poland) at the end of the surgery. After the surgery, the animals were placed individually in new home cages (43 cm × 27 cm × 18 cm; cage #IIIH UNO Life Science Solutions, Zevenaar, The Netherlands) and moved to a warm observation room until they regained consciousness. Surgery was performed on a maximum of 9 rats per day. The surgical cycle began at 8:00 a.m., and each surgery lasted approximately 90 min. Two people were responsible for the surgeries, which were conducted simultaneously at two stations. In the observation room, the animals were checked every 30 min after the surgery was completed. At 8:00 p.m., no less than two hours after the last surgery of the day, the rats were transferred to the animal facility. The surgeries on 35 rats lasted a total of 5 days. All animals survived the surgery, but in four of them, a decrease in respiratory rate was observed, which was temporarily corrected by reducing the concentration of isoflurane administered while simultaneously increasing the oxygen content in the mixture. After the surgery, a 21-day recovery period began. For the first week, the animals were inspected twice daily, at 8:00 a.m. and 3:00 p.m. The healing of the scalp surrounding the electrode base was monitored. If fresh blood appeared, the wound was cleansed with an antiseptic (Octenidini dihydrochloridum, Schülke & Mayr GmbH, Norderstedt, Germany), and an antibiotic solution (penicillin procaine, Polfa) was applied subcutaneously to the wound. Food and water consumption were also monitored during the morning observation period. All rats were drinking water and eating food without any problems the day after surgery. After a week, the wounds in all rats had healed, but the rats continued to be inspected twice daily. All 35 rats survived the recovery period, and no mobility problems or behavioral abnormalities were noted. In one rat, the tip of the anode electrode broke.

### 4.4. Electrical Stimulation of the Raphe Magnus

Preparation of rats for stimulation was carried out according to the procedure described previously [75]. However, the most important aspects are described below. After a 21-day recovery period, rats were habituated to the stimulation room and maze for one day to lower their anxiety levels. For this purpose, the animals were placed individually in a maze in which an NT had been previously performed so that they could move freely there for a period of 30 min. The next day, the behavioral effects of increasing the pre-stimulation intensity from 0 to 200 μA were examined in individual animals. Six rats were tested simultaneously, resulting in 6 groups. The first group underwent the procedure at 8:00 a.m., with subsequent groups undergoing the procedure every 45 min. During the pre-stimulation procedure, locomotor arousal as a main factor, as well as increased breathing, teeth grinding, intense sniffing, quick and repetitive movements of either the head or forelimbs, and the rat’s rotation around its own axis, were observed. The range of current intensity was set at 60–160 μA. We set the stimulation current value for each rat individually to one that was associated with locomotor arousal, while sniffing, teeth grinding, and quick and repetitive movements or body rotation suggested potential discomfort and indicated too high an intensity value. Animals that showed increased locomotor activity during pre-stimulation were assigned to a group that subsequently received electrical stimulation of the RMg (RMg-ST, n = 21). Animals that did not experience increased mobility within the administered current range or showed discomfort-like responses within this current range were assigned to a control group (RMg-Sham, n = 14). A rat that broke its electrode during recovery was also included in the control group. The following day, the 1st of the stimulation, rats was placed individually in the maze, and the cable was connected to the stimulating electrode and the anode. Again, 6 rats were tested simultaneously, resulting in 6 groups, and the first group underwent the procedure at 8:00 a.m. In each group there were animals from both the RMg-ST and RMg-Sham. The current parameters were determined according to the method described by Urayama et al. [76], with many modifications. Firstly, the frequency of stimulation was changed. The frequency was set at 130 Hz, and the pulse width at 60 μs, over the stimulation period for all the stimulated animals from the RMg-ST group. Secondly, the stimulation lasted 25 min and consisted of 30 cycles of 30 s current flow with a 20 s break between cycles. Stimuli were delivered by a stimulator unit (215/T, Hugo Sachs Elektronik GmbH, March, Germany) that provided rectangular pulses. Finally, the rats could move freely during the electrical stimulation, which allowed us to assess their mobility. The current was administered to the electrodes by a researcher who manually activated the current cycles on the stimulator unit. Simultaneously, the researcher observed the animals’ behavior, which he recorded by following a protocol, and turned off the current in the event of adverse reactions. However, such situations did not occur. The RMg-Sham group underwent the same procedure during the stimulation day, including the connection of the cable to the electrodes and staying for 25 min in the maze; however, it was not subjected to current flow through the electrode implanted in the brain (a naïve procedure). The stimulation period was planned to take place over 15 days. Firstly, the goal was to induce changes in the brain through chronic and repetitive stimulation. Secondly, we conducted behavioral tests on selected days, 30 min after the stimulation ended. To avoid stressing the animals, a three-day break was planned between the anxiety and spatial memory tests, with only stimulation being performed. A two-day break was then maintained between the spatial memory test and brain sampling for immunofluorescence assays, with only stimulation being performed.

### 4.5. Elevated Plus Maze

The EPM was performed on the 4th and 5th days of stimulation. After the RMg electrical stimulation or the naïve procedure, rats returned to their home cages. Then, after 30 min of resting, the animals were carried in their home cages to the experimental room. Finally, after 5 min of adaptation to the room, the EPM began, where measurements were taken from 9:05 a.m. to 2:15 p.m. The maze consisted of two open arms (10 cm in width and 50 cm in length) and two enclosed arms (10 cm in width, 50 cm in length, and 40 cm in height) elevated 50 cm above the floor. The maze was cleaned with 70% ethanol before the start of every trial.

Rats were placed in the maze, always in the same position, facing towards the open end of the maze, and were allowed to explore it for 5 min. Among the 35 animals, which were tested twice (70 measurements in total), there were no recorded falls from the maze. The rats’ movements were recorded using a video camera (Ikegami, Ikegami-Electronics, Neuss, Germany). The video camera was positioned approximately 250 cm over the maze’s center and connected to a video-tracking digitizing device (EthoVision XT10, Noldus, Wageningen, The Netherlands). The 1st day of measurements was treated as a stage of habituation to the experimental room and apparatus, as well as a training phase. On the 2nd day, the results were analyzed and interpreted to identify anxiety-like behaviors and the motility of the animals. Registered and analyzed parameters included the time spent in open arms, closed arms, and the maze center, as well as the number of entries into each area, as previously described [77]. In the results section, we present the motility results and the time spent by the animals in each arm of the maze. Furthermore, animals’ latency to the first movement at the beginning of the measurement period after being placed in the maze was considered an additional factor related to anxiety. None of the rats experienced freezing for longer than 1 min (20% of the measurement time); hence, all obtained results were interpreted.

### 4.6. Morris Water Maze

The MWM was used on the basis of the protocol described previously [36]. The experiment used a black circular maze (Ø 150 cm and 60 cm high) in a room illuminated by dispersed light. The maze was filled with water at a temperature of 25 °C and at a height of 25 cm. A video camera was positioned approximately 250 cm over the center of the maze and connected to a video-tracking digitizing device. The maze was virtually divided into four quadrants: north-east (NE), south-east (SE), south-west (SW), and north-west (NW). A specific external cue was provided for the duration of the MWM: a white round paper spot (Ø 10 cm) was glued to the inner surface of the black wall of the maze 10 cm above the water surface on the border of the NE and NW quadrants. This specific navigation cue was provided to compensate for impaired visual acuity in albino rat strains. The test began with a training session on the 9th day of the stimulation procedure. Spatial memory was tested on days 10 and 11 and ended with a probe (reference memory evaluation task with the platform removed) on the 12th day. A training session (two trials with the visible platform) was conducted to exclude animals with motivational or sensory–motor deficits. In the 1st trial of the screening session, a rat needed to locate the platform within 60 s; otherwise, the researcher gently guided it toward the platform. After 5 min, a 2nd screening trial was performed.

Spatial memory was tested in four 120 s trials daily with a 10 min intertrial interval for the next two days. During testing, the platform was hidden 1 cm below the water’s surface, and its position remained unchanged (center of the NE quadrant of the maze). Each rat was placed in the maze on the opposite side of the platform, facing the maze wall in the SW quadrant. Each trial ended when: (1) the animal found the platform within 120 s and remained on it for 5 s, in which case the animal was then moved from the maze to the home cage, or (2) the rat had not found the platform after 120 s, in which case the rat was navigated to the platform, left on it for 5 s, and then removed from the maze and taken to the home cage. It was decided to shorten the standard MWM protocol of measuring spatial memory to two days in order to minimize not only stress but also animal fatigue resulting from the overlap of RMg electrical stimulation or the naïve procedure.

The probe with the removed platform was performed on the 12th day of the simulation, 24 h after the last session of spatial memory measurements. One 120 s trial was conducted for each rat (starting position in the SW quadrant).

The scores used to analyze the animal’s performance in spatial memory included latency to reach the platform after placing the animal into the maze. The scores used to analyze the animal’s performance in the probe included the percentage of the total distance traveled in the critical quadrant (where the platform was located in previous days). Furthermore, the total distance traveled in the maze was analyzed as a marker of motility.

### 4.7. Brain Tissue Preparation

Sixty minutes after the end of RMg electrical stimulation (or the naïve procedure in control animals), rats were euthanized with Morbital (2 mL/kg) and transcardially perfused via the left ventricle with 200 mL of 0.9% saline, followed by 200 mL of 4% paraformaldehyde in 0.1 M phosphate-buffered saline (PBS; Biomaxima, Gdansk, Poland). The brains were removed quickly, postfixed, and cryoprotected in a 30% sucrose solution in PBS. Additionally, brain collection was performed three days after the MWM to avoid a potential influence of the behavioral test on brain changes, especially c-Fos expression. The brains were frozen and stored at −70 °C until cryostat sectioning (CM 1850, Leica Biosystems, Nussloch, Germany). Coronal 20 μm thick brain sections between 1.50 and 3.00 mm posterior to the bregma were taken. Sections including the M2 (between 1.56 and 1.64 mm, 5 slices per animal), the DG (between 2.46 and 2.54 mm, 5 slices per animal), and the ARC (between 2.86 and 2.94 mm, 5 slices per animal) were chosen for the double immunofluorescence method for 5–HT and c-Fos expression.

For Nissl staining, coronal 30 μm thick tissue sections were cut at 9.80 mm posterior to the bregma according to the rat brain stereotaxic atlas [74], containing the RMg. Sections containing RMg between 9.98 and 10.01, 10.28 and 10.31, and 10.58 and 10.61 mm (6 slices per animal) were also chosen for the double immunofluorescence method for 5–HT and c-Fos expression.

In total, 21 brain sections were subjected to immunofluorescence analysis, of which, in the case of the M2 and DG, the structures were analyzed in both hemispheres, while the ARC and RMg are located in the midline.

Histology confirmed the electrode’s localization for rats with the RMg electrical stimulation (RMg-ST) and control rats with the naïve procedure (RMg-Sham) after their sacrifice at the end of the stimulation period.

Nissl staining showed that the tips of the stimulating electrodes were localized in the RMg-ST group between 10.00 and 10.60 mm posterior to the bregma, usually in the brain midline (no more than 0.05 mm left or right) and at the right depth (10.20 mm) (Table 1).

The results of the rats in the RMg-Sham group considerably deviated from those of rats in the RMg-ST group in terms of the RMg location; in this group, it was too distant from the midline (4 rats), too shallowly implanted (4 rats), or further from bregma (3 rats) (Table 2). One rat from the RMg-ST group was added to the RMg-Sham group because of its adverse reaction to the electrical stimulation during pre-stimulation, and another one because of electrode damage during convalescence.

### 4.8. Immunofluorescence 5–HT+/c-Fos+ Imaging

Immunofluorescence processing and imaging of combined 5–HT/c-Fos were performed according to the previously described method [73] with some modifications. All steps of the double staining procedure were performed at room temperature. The sections were rinsed with PBS (pH 7.4), incubated in Normal Goat Serum (NGS, Sigma-Aldrich, St. Louis, MO, USA, containing 0.3% Triton X–100) for 30 min, and then rinsed again with PBS. Following incubation for 20 min with a blocking solution containing PBS and 0.5% Bovine Serum Albumin (BSA, Sigma-Aldrich) to reduce nonspecific binding, sections were incubated for 48 h at 4 °C in a cocktail of primary antibodies containing polyclonal rabbit anti-5–HT IgG (Abcam, Cambridge, UK; ab66047, dilution 1:300) and *c-fos* mouse monoclonal IgG (Santa Cruz Biotechnology, Dallas, TX, USA; sc–166940, dilution 1:300) diluted in PBS with 0.2% Triton X–100 and 3% NGS (Sigma-Aldrich). Following multiple rinses in 0.05 M Tris buffer (pH 7.4), the sections were incubated for 120 min in a cocktail of secondary antibodies conjugated with fluorochromes containing Alexa Fluor 488 goat anti-rabbit IgG (Invitrogen, Carlsbad, CA, USA; A11034, dilution 1:400) and Alexa Fluor 546 goat anti-mouse IgG (Invitrogen; A11030, dilution 1:400). Dilutions of primary and secondary antibodies were established according to the producers’ recommendations and our preliminary methodological trials. Autofluorescence and channel bleed-through were controlled by selecting fluorophores with well-separated emission spectra (using a red Alexa Fluor 546 for c-Fos and a green Alexa Fluor 488 for 5–HT), using optical filters with appropriate selectivity and by periodically examining unstained samples to assess background signal. The test for the specificity of an antibody (negative control) was performed by omitting the primary antibodies. All stages of immunofluorescence were identical to those described previously, except for the first incubation: sections were placed in PBS with 0.2% Triton X–100 for 48 h without primary antibodies. Negative controls were prepared periodically to confirm the absence of nonspecific fluorescence.

### 4.9. Microscopic Analysis

The staining process was completed by embedding a hardening resin (EverBrite Hardset Mounting Medium; Biotium, Fremont, CA, USA) and applying coverslips (24 mm × 60 mm, Bionovo, Legnica, Poland). Fluorescent images were taken using a PrimoStar microscope (four-channel system) (Carl Zeiss MicroImaging GmbH, Jena, Germany) (magnification 20× objective, 10× ocular). All micrographs were labeled with a white label on a black background, with a grayscale from 0 (black) to 255 (white), and processed using Carl Zeiss Imaging Systems software (Axio Vision Rel. 4.9.1; Carl Zeiss MicroImaging GmbH). For each of the analyzed sections, measurements of 2 fields were performed for the structure. The boundaries of the brain structures were determined based on the rat brain stereotaxic atlas [74] using contour traces from the templates. The software counted a defined area of brain structures, where optical density and size filters were calibrated to count white grains (>70% white) exceeding 15 pixels (15 μm^2^). Thus, the software excluded any remaining particles inconsistent with the size of the cell nucleus for c-Fos or cells containing 5–HT at a given magnification. The density of c-Fos+ nuclei, defined as the number of counted nuclei with c-Fos and cells with 5–HT per 1 mm^2^ of the surface area of the analyzed structure, was then counted. Microscopic analysis included c-Fos+ nuclei in selected forebrain and midbrain structures. Despite automatic analysis, every one of five micrographs of the fields for the structure was subjected to manual verification. Moreover, we used Nissl staining to determine the location of the stimulating electrode. The selected sections were placed on slides, stained with Cresyl violet (Sigma-Aldrich), dehydrated, and finally mounted with Distyrene, Plasticiser, Xylene medium (DPX; Sigma-Aldrich). The experimental group presented above (RMg-ST) did not include animals with misplaced electrodes. Researchers performing immunofluorescence analyses and histological verification knew only the animal number, they were not aware of the allocation to the stimulation or control group.

### 4.10. Statistical Analysis

The statistical analyses were performed using Statistica ver. 13.3 (TIBICO Software Inc., San Ramon, CA, USA). All statistical analyses were performed using standard parametric or non-parametric methods depending on the distribution of the data. Normality of each dataset was assessed using the Kolmogorov–Smirnov test with the Lilliefors correction. For normally distributed variables, comparisons between two independent groups (RMg-ST vs. RMg-Sham) were conducted using independent-samples *t*-tests. When data did not meet the assumptions of normality, the Mann–Whitney U test was used instead. Paired data obtained from repeated measurements across two consecutive days (e.g., EPM adaptation vs. test day) were evaluated using paired-samples *t*-tests or the Wilcoxon signed-rank test, depending on normality.

For experiments involving repeated trials across multiple days (e.g., locomotor activity during stimulation sessions, spatial memory performance in the MWM), a two-way ANOVA was applied with group and day or group and trial as fixed factors. When appropriate, interaction effects (group × day or group × trial) were examined. In cases where ANOVA revealed significant main or interaction effects, post hoc comparisons were performed using the Games–Howell test, which does not assume equal variances or balanced sample sizes.

Histological counts of c-Fos+ nuclei and 5–HT+ cells were analyzed using *t*-tests or Mann–Whitney U tests depending on the distribution of each dataset. For analyses comparing multiple brain regions (M2, ARC, DG, RMg), a two-way ANOVA with group and brain structure as fixed factors was conducted to assess global effects and interaction terms, followed by Games–Howell post hoc tests.

All statistical tests were two-tailed, and differences were considered statistically significant at *p* < 0.05. Data are presented as mean ± SE or median with interquartile range (IQR), depending on the distribution.

## Figures and Tables

**Table 1:** Localization of the electrode tips in the RMg in all rats with RMg electrical stimulation (RMg-ST, n = 21) according to the rat brain stereotaxic atlas [74].

Experimental Group	Distance from Bregma (mm)		
	Number of Stimulation Electrode Tips		
	–10.00	–10.30	–10.60
Rats with RMg electrical stimulation (RMg-ST; n = 21)	6	12	3

n: the number of rats in the group.

**Table 2:** Localization of the electrode tips in all rats without RMg electrical stimulation (RMg-Sham, n = 14) according to the rat brain stereotaxic atlas [74].

Control Group	Number of Electrode Tips			
	Correct Electrode Implantation	Incorrect Distance from Midline	Incorrect Implantation Depth	Incorrect Distance from Bregma
Rats without RMg electrical stimulation (RMg-Sham; n = 14)	2	4	4	3

n: the number of rats in the group.

## Data Availability

The raw data supporting the conclusions of this article will be made available by the authors on request.

## Supplementary Materials

The following supporting information can be downloaded at: https://www.mdpi.com/article/10.3390/ijms27104215/s1.

## Abbreviations

5–HT	serotonin
5–HT+	5–HT-positive cells
ARC	arcuate nucleus of the hypothalamus
B6	dorsal raphe nucleus
B8	median raphe nucleus
c-Fos+	c-Fos-positive nuclei
DG	dentate gyrus
EPM	elevated plus maze
MWM	Morris water maze
M2	secondary motor cortex
NT	novelty test
PBS	phosphate-buffered saline
RMg	raphe magnus
RMg-Sham	control group of rats with the naïve stimulation of the raphe magnus
RMg-ST	group of rats with the electrical stimulation of the raphe magnus
