# Supplementary material for: Effects of Electrical Stimulation of Raphe Magnus on Locomotion and Selected Cognitive Abilities in Rats
Source: Int J Mol Sci. 2026 May 9;27(10):4215. doi: 10.3390/ijms27104215 (PMC13207257; doi:10.3390/ijms27104215)
Supplement: Supplementary file 1 [file ijms-27-04215-s001.zip › ijms-4206452-supplementary.pdf]

Table S1. Results of the full 120-min novelty test for rats following the pre-stimulation procedure and assigned to either the RMg-ST or RMg-Sham group.
[n – number of individuals per group, K-S states from Kolmogorov-Smirnov normality test (L – Liliefors significant level); SD states from the standard deviation; SE states from the standard error; IQR states from the interquartile range; t-test states from Student’s t-test; U states from Mann-Whitney U test]

group	n	mean	median	K-S	SD	SE	IQR
RMg-Sham	14	4534.8	3812	D = 0.184<br>p > 0.20<br>L < 0.20	2077.50	555.23	2079.8
RMg-ST	21	4751.7	4913	D = 0.094<br>p > 0.20<br>L > 0.20	1505.60	328.55	2065.7

t-test	U
**t = -0.359<br>p = 0.72**	**Z = -0.791<br>p = 0.43**
df 33	U = 123.0

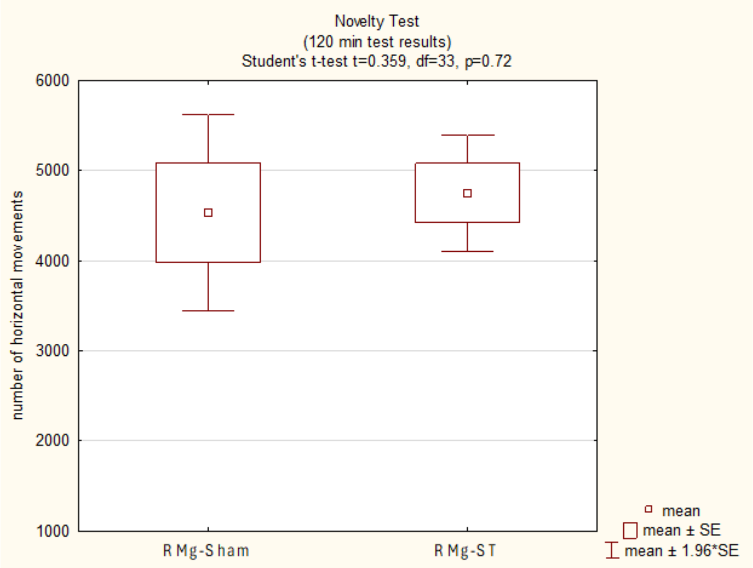

Figure S1. Comparison of rats’ locomotor activity in the novelty test (full 120 min).

Table S2. Selected results from the novelty test (first 30 min) for rats after the pre-stimulation procedure assigned to the RMg-ST or RMg-Sham groups — corresponding to Figure 1 in the manuscript.

[n – number of individuals per group, K-S states from Kolmogorov-Smirnov normality test (L – Liliefors significant level); SD states from the standard deviation; SE states from the standard error; IQR states from the interquartile range; t-test states from Student's t-test; U states from Mann-Whitney U test]

group	n	mean	median	K-S	SD	SE	IQR
RMg-Sham	14	1839.8	1725	D = 0.125<br>p > 0.20	619.66	166.61	990.2
				L > 0.20			
RMg-ST	21	1798.7	1770	D = 0.101<br>p > 0.20	606.75	132.40	1010.0
				L > 0.20			

t-test	U
**t = 0.195**<br>**p = 0.85**<br>df 33	**Z = 0.118**<br>**p = 0.91**<br>U = 143.0

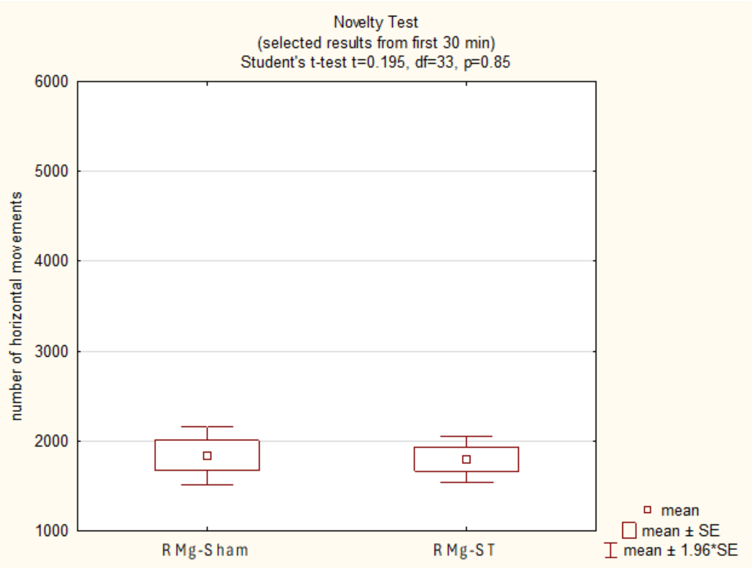

Figure S2. Comparison of rats' locomotor activity in the novelty test (first 30 min after the test start).

Table S3. Results of locomotor activity in rats during RMg electrical stimulation (RMg-ST) or a naïve procedure in control animals (RMg-Sham) across stimulation days — corresponding to Figure 2 in the manuscript.
[n – number of individuals per group, K-S states from Kolmogorov-Smirnov normality test (L – Liliefors significant level); SD states from the standard devia-tion; SE states from the standard error; IQR states from the interquartile range; t-test states from Student's t-test; U states from Mann-Whitney U test]

day	group	n	mean	median	K-S	SD	SE	IQR	t-test	U
1	RMg-Sham	14	593.1	350	D = 0.221<br>p > 0.20	592.2	158.28	659.0	**t = -3.733<br>p < 0.001**	**Z = -4.259<br>p < 0.000**
					L < 0.10				df 33	U = 20.0
	RMg-ST	21	4310.1	3006	D = 0.202<br>p > 0.20	3676.1	802.2	5173.0		
					L < 0.05					
2	RMg-Sham	14	644.9	215	D = 0.270<br>p > 0.20	920.7	246.1	719.0	**t =-4.514<br>p < 0.000**	**Z = -4.091<br>p < 0.000**
					L < 0.01				df 33	U = 25.0
	RMg-ST	21	5114.1	5216	D = 0.114<br>p > 0.20	3610.0	787.8	5598.0		
					L > 0.20					
3	RMg-Sham	14	579.1	240	D = 0.299<br>p < 0.15	792.6	211.8	357.2	**t = -4.521<br>p < 0.000**	**Z = -4.462<br>p < 0.000**
					L < 0.01				df 33	U = 14.0
	RMg-ST	21	3716.8	3506	D = 0.200<br>p > 0.20	2503.5	546.3	1870.0		
					L < 0.05					
4	RMg-Sham	14	425.6	202	D = 0.243<br>p > 0.20	537.5	143.6	384.0	**t =-5.272<br>p < 0.000**	**Z = -4.698<br>p < 0.000**
					L < 0.05				df 33	U = 7.0
	RMg-ST	21	4101.1	4012	D = 0.163<br>p > 0.20	2559.1	558.4	2599.0		
					L < 0.15					
5	RMg-Sham	14	344.7	192	D = 0.284<br>p < 0.20	424.6	113.5	265.5	**t = -5.601<br>p < 0.000**	**Z = -4.731<br>p < 0.000**
					L < 0.01				df 33	U = 6,0
	RMg-ST	21	3037.2	3180	D = 0.176<br>p > 0.20	1756.7	383.4	2607.0		
					L < 0.10					
6	RMg-Sham	14	296.9	115	D = 0.281<br>p < 0.20	318.4	85.2	380.8	**t = -4.787<br>p < 0.000**	**Z = -4.901<br>p < 0.000**
					L < 0.01				df 33	U = 1.0
	RMg-ST	21	3603.5	3053	D = 0.223<br>p > 0.15	2558.7	558.4	2315.0		
					L < 0.01					
7	RMg-Sham	14	291.6	163	D = 0.310<br>p < 0.15	321.9	86.0	166.8	**t = -4.861<br>p < 0.000**	**Z = -4.698<br>p < 0.000**
					L < 0.01				df 33	U = 7.0
	RMg-ST	21	3040.5	2436	D = 0.156<br>p > 0.20	2089.1	455.9	2728.0		
					L < 0.15					

8	RMg-Sham	14	317.5	153	D = 0.273<br>p > 0.20	297.9	79.6	290.8	**t = -6.048<br>p < 0.000**	**Z = -4.866<br>p < 0.000**
					L < 0.01				df 33	U = 2.0
	RMg-ST	21	3340.1	3297	D = 0.116<br>p > 0.20	1845.0	402.6	2166.0		
					L > 0.20					
9	RMg-Sham	14	364.3	140	D = 0.272<br>p > 0.20	436.3	116.6	324.0	**t = -6.036<br>p < 0.000**	**Z = -4.665<br>p < 0.000**
					L < 0.01				df 33	U = 8.0
	RMg-ST	21	3198.0	3260	D = 0.138<br>p > 0.20	1711.9	373.6	2876.0		
					L > 0.20					
10	RMg-Sham	14	454.2	309	D = 0.290<br>p < 0.20	340.9	91.1	481.2	**t =-5.808<br>p < 0.000**	**Z=-4.933<br>p < 0.000**
					L < 0.01				df 33	U = 0.0
	RMg-ST	21	3422.7	3213	D = 0.188<br>p > 0.20	1882.9	410.9	2891.0		
					L < 0.05					
11	RMg-Sham	14	475.9	370	D = 0.161<br>p > 0.20	338.9	90.6	390.5	**t = -5.234<br>p < 0.000**	**Z = -4.866<br>p < 0.000**
					L > 0.20				df 33	U = 2.0
	RMg-ST	21	3751.1	3307	D = 0.129<br>p > 0.20	2313.5	504.9	3396.0		
					L > 0.20					
12	RMg-Sham	14	388.1	252	D = 0.205<br>p > 0.20	333.0	89.0	344.8	**t = -6.333<br>p < 0.000**	**Z = -4.933<br>p < 0.000**
					L < 0.15				df 33	U = 0.0
	RMg-ST	21	3382.9	3641	D = 0.208<br>p > 0.20	1740.0	379.7	2642.0		
					L < 0.01					
13	RMg-Sham	14	336.9	172	D = 0.227<br>p > 0.20	433.3	115.8	475.0	**t = -5.363<br>p < 0.000**	**Z = -4,664<br>p < 0,000**
					L < 0.05				df 33	U = 8.0
	RMg-ST	21	2979.0	2441	D = 0.208<br>p > 0.20	1800.6	392.9	1263.0		
					L < 0.01					
14	RMg-Sham	14	203.0	79	D = 0.361<br>p < 0.05	261.6	69.9	101.2	**t = -6.300<br>p < 0.000**	**Z = -4.934<br>p < 0.000**
					L < 0.01				df 33	U = 0.0
	RMg-ST	21	3069.6	2534	D = 0.188<br>p > 0.20	1680.9	366.8	2401.0		
					L < 0.05					
15	RMg-Sham	14	214.5	131	D = 0.296<br>p < 0.15	187.0	50.0	241.4	**t = -8.476<br>p < 0.000**	**Z = -4.933<br>p < 0.000**
					L < 0.01				df 33	U = 0.0
	RMg-ST	21	3276.5	3415	D = 0.171<br>p > 0.20	1336.4	291.6	1760.0		
					L < 0.10					

Table S4. Results of the two-way ANOVA, including tests of significance, effect sizes, and statistical power, for mobility.

Effects	SS	degrees of freedom	MS	F	p
Intercept	2.0E+9	1	2.0E+09	596.078	0.00
group	1.3E+09	1	1.3E+09	381.394	0.00
day	5.7E+09	14	4.1E+09	1.229	0.25
group × day	2.7E+09	14	2.0E+09	6.594	0.02
Error	1.6E+09	495	3.3E+09		

Table S5. Results of Levene's test for homogeneity of variance for the group × day effects.

Effects	SS	degrees of freedom	MS	F	p
Intercept	1.2E+07	1	1.2E+06	9.583	0.00

Table S6. Games–Howell post-hoc analysis of the group × day effects.

	group	day	{1}	{2}	{3}	{4}	{5}	{6}	{7}	{8}	{9}	{10}	{11}	{12}	{13}	{14}	{15}	{16}	{17}	{18}	{19}	{20}	{21}	{22}	{23}	{24}	{25}	{26}	{27}	{28}	{29}	{30}
			593	645	579	426	345	297	292	318	318	454	476	388	337	203	214	4310	5114	3717	4101	3037	3604	3040	3340	3340	3423	3751	3383	2979	3070	3276
1	RMg-Sham	1		0,94	0,98	0,81	0,72	0,67	0,66	0,69	0,69	0,84	0,86	0,77	0,71	0,57	0,58	0,00	0,00	0,00	0,00	0,09	0,00	0,09	0,02	0,02	0,01	0,00	0,02	0,12	0,08	0,03
2		2	0,94		0,92	0,75	0,66	0,61	0,61	0,63	0,63	0,78	0,81	0,71	0,65	0,52	0,53	0,00	0,00	0,00	0,00	0,12	0,01	0,12	0,03	0,03	0,02	0,00	0,02	0,15	0,10	0,04
3		3	0,98	0,92		0,82	0,73	0,68	0,68	0,70	0,70	0,86	0,88	0,78	0,73	0,59	0,60	0,00	0,00	0,00	0,00	0,09	0,00	0,09	0,02	0,02	0,01	0,00	0,02	0,12	0,08	0,03
4		4	0,81	0,75	0,82		0,91	0,85	0,85	0,88	0,88	0,97	0,94	0,96	0,90	0,75	0,76	0,00	0,00	0,00	0,00	0,04	0,00	0,04	0,01	0,01	0,00	0,00	0,01	0,06	0,04	0,01
5		5	0,72	0,66	0,73	0,91		0,94	0,94	0,97	0,97	0,87	0,85	0,95	0,99	0,84	0,85	0,00	0,00	0,00	0,00	0,03	0,00	0,03	0,00	0,00	0,00	0,00	0,00	0,04	0,02	0,01
6		6	0,67	0,61	0,68	0,85	0,94		0,99	0,98	0,98	0,82	0,79	0,89	0,95	0,89	0,90	0,00	0,00	0,00	0,00	0,02	0,00	0,02	0,00	0,00	0,00	0,00	0,00	0,03	0,02	0,01
7		7	0,66	0,61	0,68	0,85	0,94	0,99		0,97	0,97	0,81	0,79	0,89	0,95	0,90	0,91	0,00	0,00	0,00	0,00	0,02	0,00	0,02	0,00	0,00	0,00	0,00	0,00	0,03	0,02	0,01
8		8	0,69	0,63	0,70	0,88	0,97	0,98	0,97		1,00	0,84	0,82	0,92	0,98	0,87	0,88	0,00	0,00	0,00	0,00	0,02	0,00	0,02	0,00	0,00	0,00	0,00	0,00	0,03	0,02	0,01
9		9	0,69	0,63	0,70	0,88	0,97	0,98	0,97	1,00		0,84	0,82	0,92	0,98	0,87	0,88	0,00	0,00	0,00	0,00	0,02	0,00	0,02	0,00	0,00	0,00	0,00	0,00	0,03	0,02	0,01
10		10	0,84	0,78	0,86	0,97	0,87	0,82	0,81	0,84	0,84		0,97	0,92	0,86	0,72	0,73	0,00	0,00	0,00	0,00	0,05	0,00	0,05	0,01	0,01	0,01	0,00	0,01	0,07	0,04	0,01
11		11	0,86	0,81	0,88	0,94	0,85	0,79	0,79	0,82	0,82	0,97		0,90	0,84	0,69	0,70	0,00	0,00	0,00	0,00	0,05	0,00	0,05	0,01	0,01	0,01	0,00	0,01	0,07	0,05	0,02
12		12	0,77	0,71	0,78	0,96	0,95	0,89	0,89	0,92	0,92	0,92	0,90		0,94	0,79	0,80	0,00	0,00	0,00	0,00	0,04	0,00	0,03	0,01	0,01	0,00	0,00	0,00	0,05	0,03	0,01
13		13	0,71	0,65	0,73	0,90	0,99	0,95	0,95	0,98	0,98	0,86	0,84	0,94		0,85	0,86	0,00	0,00	0,00	0,00	0,03	0,00	0,03	0,00	0,00	0,00	0,00	0,00	0,04	0,02	0,01
14		14	0,57	0,52	0,59	0,75	0,84	0,89	0,90	0,87	0,87	0,72	0,69	0,79	0,85		0,99	0,00	0,00	0,00	0,00	0,01	0,00	0,01	0,00	0,00	0,00	0,00	0,00	0,02	0,01	0,00
15		15	0,58	0,53	0,60	0,76	0,85	0,90	0,91	0,88	0,88	0,73	0,70	0,80	0,86	0,99		0,00	0,00	0,00	0,00	0,01	0,00	0,01	0,00	0,00	0,00	0,00	0,00	0,02	0,01	0,00
16	RMg-ST	1	0,00	0,00	0,00	0,00	0,00	0,00	0,00	0,00	0,00	0,00	0,00	0,00	0,00	0,00	0,00		0,15	0,29	0,71	0,02	0,21	0,02	0,09	0,09	0,12	0,32	0,10	0,02	0,03	0,07
17		2	0,00	0,00	0,00	0,00	0,00	0,00	0,00	0,00	0,00	0,00	0,00	0,00	0,00	0,00	0,00	0,15		0,01	0,07	0,00	0,01	0,00	0,00	0,00	0,00	0,02	0,00	0,00	0,00	0,00
18		3	0,00	0,00	0,00	0,00	0,00	0,00	0,00	0,00	0,00	0,00	0,00	0,00	0,00	0,00	0,00	0,29	0,01		0,49	0,23	0,84	0,23	0,50	0,50	0,60	0,95	0,55	0,19	0,25	0,43
19		4	0,00	0,00	0,00	0,00	0,00	0,00	0,00	0,00	0,00	0,00	0,00	0,00	0,00	0,00	0,00	0,71	0,07	0,49		0,06	0,38	0,06	0,18	0,18	0,23	0,53	0,20	0,05	0,07	0,14
20		5	0,09	0,12	0,09	0,04	0,03	0,02	0,02	0,02	0,02	0,05	0,05	0,04	0,03	0,01	0,01	0,02	0,00	0,23	0,06		0,31	1,00	0,59	0,59	0,49	0,20	0,54	0,92	0,95	0,67
21		6	0,00	0,01	0,00	0,00	0,00	0,00	0,00	0,00	0,00	0,00	0,00	0,00	0,00	0,00	0,00	0,21	0,01	0,84	0,38	0,31		0,32	0,64	0,64	0,75	0,79	0,69	0,27	0,34	0,56
22		7	0,09	0,12	0,09	0,04	0,03	0,02	0,02	0,02	0,02	0,05	0,05	0,03	0,03	0,01	0,01	0,02	0,00	0,23	0,06	1,00	0,32		0,59	0,59	0,50	0,21	0,54	0,91	0,96	0,67
23		8	0,02	0,03	0,02	0,01	0,00	0,00	0,00	0,00	0,00	0,01	0,01	0,01	0,00	0,00	0,00	0,09	0,00	0,50	0,18	0,59	0,64	0,59		1,00	0,88	0,46	0,94	0,52	0,63	0,91
24		9	0,02	0,03	0,02	0,01	0,00	0,00	0,00	0,00	0,00	0,01	0,01	0,01	0,00	0,00	0,00	0,09	0,00	0,50	0,18	0,59	0,64	0,59	1,00		0,88	0,46	0,94	0,52	0,63	0,91
25		10	0,01	0,02	0,01	0,00	0,00	0,00	0,00	0,00	0,00	0,01	0,01	0,00	0,00	0,00	0,00	0,12	0,00	0,60	0,23	0,49	0,75	0,50	0,88	0,88		0,56	0,94	0,43	0,53	0,79
26		11	0,00	0,00	0,00	0,00	0,00	0,00	0,00	0,00	0,00	0,00	0,00	0,00	0,00	0,00	0,00	0,32	0,02	0,95	0,53	0,20	0,79	0,21	0,46	0,46	0,56		0,51	0,17	0,23	0,40
27		12	0,02	0,02	0,02	0,01	0,00	0,00	0,00	0,00	0,00	0,01	0,01	0,00	0,00	0,00	0,00	0,10	0,00	0,55	0,20	0,54	0,69	0,54	0,94	0,94	0,94	0,51		0,47	0,58	0,85
28		13	0,12	0,15	0,12	0,06	0,04	0,03	0,03	0,03	0,03	0,07	0,07	0,05	0,04	0,02	0,02	0,02	0,00	0,19	0,05	0,92	0,27	0,91	0,52	0,52	0,43	0,17	0,47		0,87	0,60
29		14	0,08	0,10	0,08	0,04	0,02	0,02	0,02	0,02	0,02	0,04	0,05	0,03	0,02	0,01	0,01	0,03	0,00	0,25	0,07	0,95	0,34	0,96	0,63	0,63	0,53	0,23	0,58	0,87		0,71
30		15	0,03	0,04	0,03	0,01	0,01	0,01	0,01	0,01	0,01	0,01	0,02	0,01	0,01	0,00	0,00	0,07	0,00	0,43	0,14	0,67	0,56	0,67	0,91	0,91	0,79	0,40	0,85	0,60	0,71	

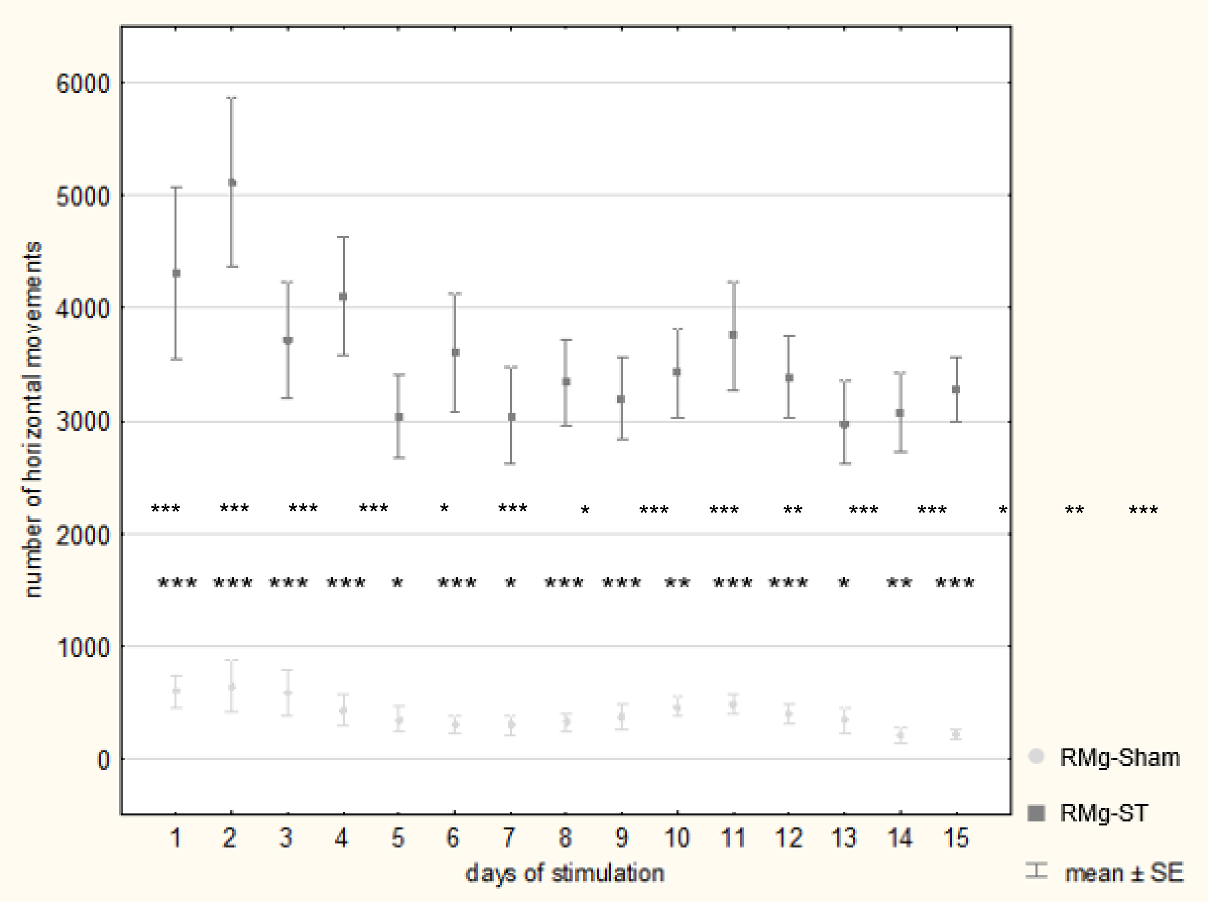

Figure S3. Comparison of locomotor activity in rats during RMg electrical stimulation (RMg-ST) or a naïve procedure in control rats (RMg-Sham).
A two-way ANOVA indicates a significant interaction between stimulation group and day ($F_{(14, 495)} = 6.594$, $p = 0.02$). Post-hoc comparisons reveal highly significant differences between groups (* $p < 0.05$; ** $p< 0.01$; *** $p < 0.001$).

Table S7. Results of paired-samples t-test – comparing consecutive days within the RMg-ST group.

RMg-ST	1	2	3	4	5	6	7	8	9	10	11	12	13	14	15
1		t=-2.010<br>p=0.06	t=1.013<br>p=0.32	t=0.441<br>p=0.32	t=2.077<br>p=0.05	t=1.090<br>p=0.29	t=1.398<br>p=0.18	t=1.634<br>p=0.12	t=1.620<br>p=0.12	t=1.212<br>p=0.24	t=0.898<br>p=0.38	t=1.357<br>p=0.19	t=1.624<br>p=0.12	t=1.705<br>p=0.10	t=1.484<br>p=0.15
2	t=-2.010<br>p=0.06		t=3.111<br>p=0.01	t=2.475<br>p=0.02	t=3.554<br>p=0.00	t=3.006<br>p=0.01	t=2.533<br>p=0.02	t=3.166<br>p=0.00	t=3.058<br>p=0.01	t=2.642<br>p=0.02	t=2.335<br>p=0.03	t=3.119<br>p=0.01	t=2.842<br>p=0.01	t=2.834<br>p=0.01	t=2.772<br>P=0.01
3	t=1.013<br>p=0.32	t=3.111<br>p=0.01		t=-0.832<br>p=0.42	t=1.589<br>p=0.13	t=0.392<br>p=0.70	t=0.981<br>p=0.34	t=0.819<br>p=0.42	t=0.980<br>p=0.34	t=0.605<br>p=0.55	t=-0.071<br>p=0.94	t=0.836<br>p=0.41	t=1.265<br>p=0.22	t=1.113<br>p=0.22	t=0.887<br>p=0.39
4	t=0.441<br>p=0.32	t=2.475<br>p=0.02	t=-0.832<br>p=0.42		t=2.421<br>p=0.03	t=0.975<br>p=0.34	t=1.574<br>p=0.13	t=2.049<br>p=0.05	t=2.196<br>p=0.04	t=1.316<br>p=0.20	t=-0.722<br>p=0.48	t=1.721<br>p=0.10	t=1.791<br>p=0.09	t=1.786<br>p=0.09	t=1.701<br>p=0.10
5	t=2.077<br>p=0.05	t=3.554<br>p=0.00	t=1.589<br>p=0.13	t=2.421<br>p=0.03		t=-1.302<br>p=0.21	t=-0.008<br>p=0.99	t=-0.845<br>p=0.41	t=-0.586<br>p=0.56	t=-1.381<br>p=0.18	t=-2.398<br>p=0.03	t=-0.931<br>p=0.36	t=0.134<br>p=0.89	t=-0.091<br>p=0.93	t=-0.830<br>p=0.42
6	t=1.090<br>p=0.29	t=3.006<br>p=0.01	t=0.392<br>p=0.70	t=0.975<br>p=0.34	t=-1.302<br>p=0.21		t=0.824<br>p=0,42	t=-0.479<br>p=0.64	t=-0.706<br>p=0.49	t=0.339<br>p=0.74	t=-0.259<br>p=0.80	t=0.517<br>p=0.61	t=1.087<br>p=0.29	t=0.867<br>p=0.40	t=0.628<br>p=0.54
7	t=1.398<br>p=0.18	t=2.533<br>p=0.02	t=0.981<br>p=0.34	t=1.574<br>p=0.13	t=-0.008<br>p=0.99	t=0.824<br>p=0.42		t=-0.665<br>p=0.51	t=-0.433<br>p=0.67	t=-1.183<br>p=0.25	t=-1.637<br>p=-0.12	t=-0.769<br>p=0.45	t=0.146<br>p=0.89	t=-0.080<br>p=0.94	t=-0.757<br>p=0.46
8	t=1.634<br>p=0.12	t=3.166<br>p=0.00	t=0.819<br>p=0.42	t=2.049<br>p=0.05	t=-0.845<br>p=0.41	t=-0.479<br>p=0.64	t=-0.665<br>p=0.51		t=-0.551<br>p=0.59	t=0.241<br>p=0.81	t=-1.267<br>p=0.22	t=-0.124<br>p=0.90	t=0.714<br>p=0.48	t=0.672<br>p=0.51	t=0.206<br>p=0.84
9	t=1.620<br>p=0.12	t=3.058<br>p=0.01	t=0.980<br>p=0.34	t=2.196<br>p=0.04	t=-0.586<br>p=0.56	t=-0.706<br>p=0.49	t=-0.433<br>p=0.67	t=-0.551<br>p=0.59		t=-0.961<br>p=0.35	t=-1.760<br>p=0.09	t=-0.491<br>p=0.63	t=0.477<br>p=0.64	t=0.365<br>p=0.72	t=-0.285<br>p=0.78
10	t=1.212<br>p=0.24	t=2.642<br>p=0.02	t=0.605<br>p=0.55	t=1.316<br>p=0.20	t=-1.381<br>p=0.18	t=0.339<br>p=0.74	t=-1.183<br>p=0.25	t=0.241<br>p=0.81	t=-0.961<br>p=0.35		t=-1.377<br>p=0.18	t=0.130<br>p=0.90	t=1.130<br>p=0.27	t=1.122<br>p=0.28	t=0.680<br>p=0.50
11	t=0.898<br>p=0.38	t=2.335<br>p=0.03	t=-0.071<br>p=0.94	t=-0.722<br>p=0.48	t=-2.398<br>p=0.03	t=-0.259<br>p=0.80	t=-1.637<br>p=-0.12	t=-1.267<br>p=0.22	t=-1.760<br>p=0.09	t=-1.377<br>p=0.18		t=0.987<br>p=0.34	t=1.757<br>p=0.09	t=2.160<br>p=0.04	t=1.555<br>p=0.14
12	t=1.357<br>p=0.19	t=3.119<br>p=0.01	t=0.836<br>p=0.41	t=1.721<br>p=0.10	t=-0.931<br>p=0.36	t=0.517<br>p=0.61	t=-0.769<br>p=0.45	t=-0.124<br>p=0.90	t=-0.491<br>p=0.63	t=0.130<br>p=0.90	t=0.987<br>p=0.34		t=1.148<br>p=0.26	t=0.824<br>p=0.42	t=0.420<br>p=0.68
13	t=1.624<br>p=0.12	t=2.842<br>p=0.01	t=1.265<br>p=0.22	t=1.791<br>p=0.09	t=0.134<br>p=0.89	t=1.087<br>p=0.29	t=0.146<br>p=0.89	t=0.714<br>p=0.48	t=0.477<br>p=0.64	t=1.130<br>p=0.27	t=1.757<br>p=0.09	t=1.148<br>p=0.26		t=-0.426<br>p=0.67	t=-1.017<br>p=0.32
14	t=1.705<br>p=0.10	t=2.834<br>p=0.01	t=1.113<br>p=0.22	t=1.786<br>p=0.09	t=-0.091<br>p=0.93	t=0.867<br>p=0.40	t=-0.080<br>p=0.94	t=0.672<br>p=0.51	t=0.365<br>p=0.72	t=1.122<br>p=0.28	t=2.160<br>p=0.04	t=0.824<br>p=0.42	t=-0.426<br>p=0.67		t=-0.971<br>p=0.34
15	t=1.484<br>p=0.15	t=2.772<br>P=0.01	t=0.887<br>p=0.39	t=1.701<br>p=0.10	t=-0.830<br>p=0.42	t=0.628<br>p=0.54	t=-0.757<br>p=0.46	t=0.206<br>p=0.84	t=-0.285<br>p=0.78	t=0.680<br>p=0.50	t=1.555<br>p=0.14	t=0.420<br>p=0.68	t=-1.017<br>p=0.32	t=-0.971<br>p=0.34	

Table S8. Results of the paired-samples t-test comparing consecutive days within the RMg-Sham group.

RMg-Sham	1	2	3	4	5	6	7	8	9	10	11	12	13	14	15
1		t=-0.437 p=0.67	t=0.131 p=0.13	t=1.350 p=0.20	t=3.455 p=0.00	t=2.481 p=0.03	t=2.085 p=0.06	t=2.429 p=0.03	t=1.519 p=0.15	t=1.367 p=0.19	t=0.938 p=0.37	t=2.187 p=0.05	t=4.127 p=0.00	t=3.884 p=0.00	t=3.161 p=0.01
2	t=-0.437 p=0.67		t=0.808 p=0.43	t=1.257 p=0.23	t=2.022 p=0.23	t=1.558 p=0.14	t=1.443 p=0.17	t=1.542 p=0.15	t=1.116 p=0.28	t=0.963 p=0.35	t=0.776 p=0.45	t=1.346 p=0.20	t=2.242 p=0.04	t=2.319 p=0.04	t=2.069 P=0.06
3	t=0.131 p=0.13	t=0.808 p=0.43		t=1.006 p=0.33	t=1.902 p=0.08	t=1.511 p=0.15	t=1.372 p=0.19	t=1.509 p=0.16	t=0.984 p=0.34	t=0.783 p=0.45	t=0.591 p=0.56	t=1.252 p=0.23	t=2.297 p=0.04	t=2.398 p=0.03	t=2.023 p=0.06
4	t=1.350 p=0.20	t=1.257 p=0.23	t=1.006 p=0.33		t=0.665 p=0.52	t=0.976 p=0.35	t=1.055 p=0.31	t=1.008 p=0.33	t=0.374 p=0.71	t=-0.280 p=0.78	t=-0.451 p=0.66	t=0.302 p=0.77	t=0.937 p=0.37	t=1.838 p=0.09	t=1.961 p=0.07
5	t=3.455 p=0.00	t=2.022 p=0.23	t=1.902 p=0.08	t=0.665 p=0.52		t=0.510 p=0.62	t=0.448 p=0.66	t=0.305 p=0.77	t=-0.141 p=0.89	t=-1.284 p=0.22	t=-1.432 p=0.18	t=-0.737 p=0.47	t=0.186 p=0.86	t=2.814 p=0.01	t=1.684 p=0.12
6	t=2.481 p=0.03	t=1.558 p=0.14	t=1.511 p=0.15	t=0.976 p=0.35	t=0.510 p=0.62		t=0.104 p=0.92	t=-0.661 p=0.52	t=-0.961 p=0.35	t=-3.418 p=0.00	t=-3.195 p=0.01	t=-1.941 p=0.07	t=-0.432 p=0.67	t=1.523 p=0.15	t=1.232 p=0.24
7	t=2.085 p=0.06	t=1.443 p=0.17	t=1.372 p=0.19	t=1.055 p=0.31	t=0.448 p=0.66	t=0.104 p=0,92		t=-0.606 p=0.55	t=-0.675 p=0.51	t=-2.166 p=0.05	t=-3.251 p=-0.01	t=-1.125 p=0.28	t=-0.384 p=0.71	t=1.123 p=0.28	t=1.123 p=0.28
8	t=2.429 p=0.03	t=1.542 p=0.15	t=1.509 p=0.16	t=1.008 p=0.33	t=0.305 p=0.77	t=-0.661 p=0.52	t=-0.606 p=0.55		t=-0.523 p=0.61	t=-3.751 p=0.00	t=-3.811 p=0.00	t=-1.386 p=0.19	t=-0.240 p=0.81	t=2.098 p=0.06	t=1.888 p=0.08
9	t=1.519 p=0.15	t=1.116 p=0.28	t=0.984 p=0.34	t=0.374 p=0.71	t=-0.141 p=0.89	t=-0.961 p=0.35	t=-0.675 p=0.51	t=-0.523 p=0.61		t=-1.181 p=0.26	t=-1.006 p=0.33	t=-0.272 p=0.79	t=0.212 p=0.84	t=1.381 p=0.19	t=1.306 p=0.21
10	t=1.367 p=0.19	t=0.963 p=0.35	t=0.783 p=0.45	t=-0.280 p=0.78	t=-1.284 p=0.22	t=-3.418 p=0.00	t=-2.166 p=0.05	t=-3.751 p=0.00	t=-1.181 p=0.26		t=-0.403 p=0.69	t=1.643 p=0.12	t=1.710 p=0.11	t=3.900 p=0.00	t=3.600 p=0,00
11	t=0.938 p=0.37	t=0.776 p=0.45	t=0.591 p=0.56	t=-0.451 p=0.66	t=-1.432 p=0.18	t=-3.195 p=0.01	t=-3.251 p=-0.01	t=-3.811 p=0.00	t=-1.006 p=0.33	t=-0.403 p=0.69		t=1.515 p=0.15	t=1.515 p=0.15	t=4.361 p=0.00	t=3.984 p=0.00
12	t=2.187 p=0.05	t=1.346 p=0.20	t=1.252 p=0.23	t=0.302 p=0.77	t=-0.737 p=0.47	t=-1.941 p=0.07	t=-1.125 p=0.28	t=-1.386 p=0.19	t=-0.272 p=0.79	t=1.643 p=0.12	t=1.515 p=0.15		t=0.839 p=0.42	t=4.318 p=0.00	t=2.672 p=0.02
13	t=4.127 p=0.00	t=2.242 p=0.04	t=2.297 p=0.04	t=0.937 p=0.37	t=0.186 p=0.86	=-0.432 p=0.67	t=-0.384 p=0.71	t=-0.240 p=0.81	t=0.212 p=0.84	t=1.710 p=0.11	t=1.515 p=0.15	t=0.839 p=0.42		t=2.141 p=0.05	t=1.529 p=0.15
14	t=3.884 p=0.00	t=2.319 p=0.04	t=2.398 p=0.03	t=1.838 p=0.09	t=2.814 p=0.01	t=1.523 p=0.15	t=1.123 p=0.28	t=2.098 p=0.06	t=1.381 p=0.19	t=3.900 p=0.00	t=4.361 p=0.00	t=4.318 p=0.00	t=2.141 p=0.05		t=-0.283 p=0.78
15	t=3.161 p=0.01	t=2.069 P=0.06	t=2.023 p=0.06	t=1.961 p=0.07	t=1.684 p=0.12	t=1.232 p=0.24	t=1.123 p=0.28	t=1.888 p=0.08	t=1.306 p=0.21	t=3.600 p=0,00	t=3.984 p=0.00	t=2.672 p=0.02	t=1.529 p=0.15	t=-0.283 p=0.78	

Table S9. Results for the distance travelled by rats in the EPM following RMg electrical stimulation (RMg-ST) or a naïve procedure in control rats (RMg-Sham) – corresponding to Figure 3A in the manuscript.

[n – number of individuals per group, K-S states from Kolmogorov-Smirnov normality test (L – Liliefors significant level); SD states from the standard deviation; SE states from the standard error; IQR states from the interquartile range; t-test states from Student's t-test; U states from Mann-Whitney U test]

distance in the maze

day	group	n	mean	median	K-S	SD	SE	IQR	t-test	U
1	RMg-Sham	14	668.3	673	D = 0.169<br>p > 0.20	129.34	34.57	127.9	**t = -4.098<br>p < 0.000**	**Z = -3.519<br>p < 0.000**
					L > 0.20				df 33	U = 42.0
	RMg-ST	21	949.7	991	D = 0.120<br>p > 0.20	233.41	50.93	320.4		
					L > 0.20					
2	RMg-Sham	14	889.7	889	D = 0.120<br>p > 0.20	100.12	26.76	146.5	**t =-6.495<br>p < 0.000**	**Z = -4.495<br>p < 0.000**
					L > 0.20				df 33	U = 13.0
	RMg-ST	21	1258.5	1267	D = 0.119<br>p > 0.20	195.40	42.64	240.6		
					L > 0.20					

Table S10. Results of the paired-samples t-test comparing consecutive days for the distance travelled by rats in the EPM.

RMg-Sham	**t = -6.131**<br>**p < 0.000**	RMg-ST	**t = -9.270**<br>**p < 0.000**
	df 13		df 20

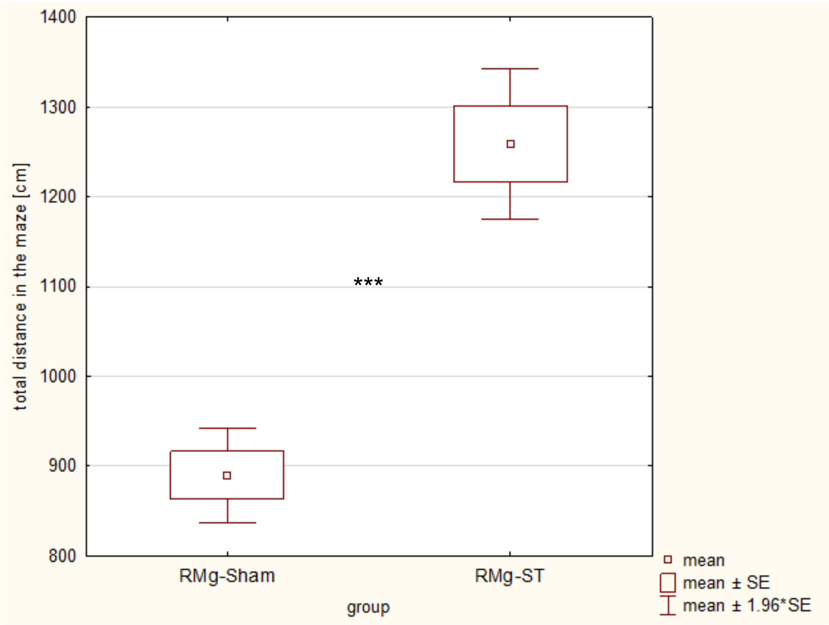

Figure S4. Comparison of the distance travelled by rats on 2nd day of testing in the EPM.
The t-test indicates a significant difference between groups (*** $p < 0.001$) in the time spent in the open arms.

Table S11. Results for the duration of movement of rats in the EPM following RMg electrical stimulation (RMg-ST) or a naïve procedure in control rats (RMg-Sham) animals – corresponding to Figure 3B in the manuscript.

[n – number of individuals per group, K-S states from Kolmogorov-Smirnov normality test (L – Liliefors significant level); SD states from the standard deviation; SE states from the standard error; IQR states from the interquartile range; t-test states from Student's t-test; U states from Mann-Whitney U test]

duration of the movement

day	group	n	mean	median	K-S	SD	SE	IQR	t-test	U
1	RMg-Sham	14	101.2	100	D = 0.146<br>p > 0.20	22.97	6.14	20.3	**t = -5.231<br>p < 0.000**	**Z = -3.014<br>p < 0.01**
					L > 0.20				df 33	U = 57.0
	RMg-ST	21	129.9	129	D = 0.089<br>p > 0.20	26.76	5.84	35.5		
					L > 0.20					
2	RMg-Sham	14	134.9	137	D = 0.107<br>p > 0.20	21.59	5.77	31.5	**t =-3.280<br>p < 0.001**	**Z = -3.855<br>p < 0.000**
					L > 0.20				df 33	U = 32.0
	RMg-ST	21	173.0	171	D = 0.127<br>p > 0.20	20.80	4.54	30.7		
					L > 0.20					

Table S12. Results of the paired-samples t-test comparing consecutive days of the duration of movement of rats in the EPM.

RMg-Sham	**t = -5.955 p < 0.000**	RMg-ST	**t = -8.796 p < 0.000**
	df 13		df 20

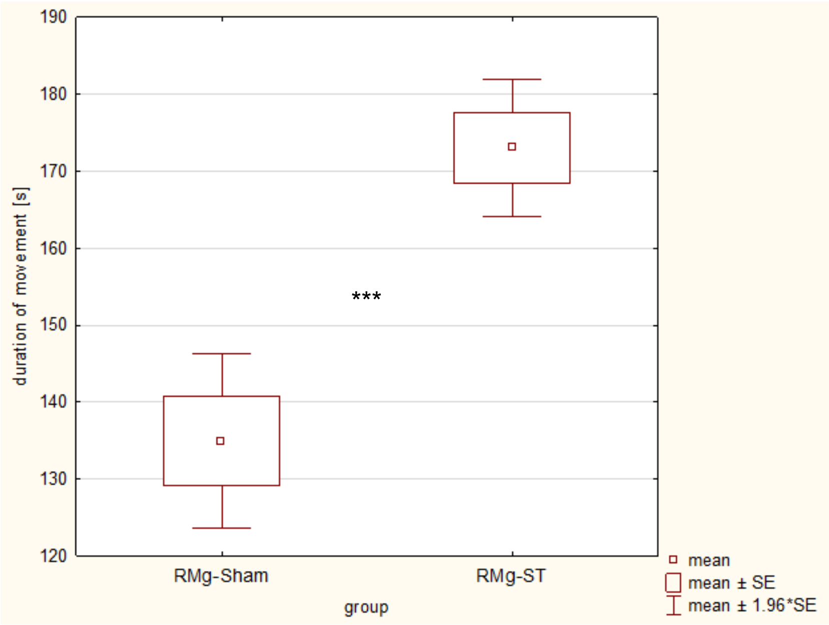

Figure S5. Comparison of the duration of movement of rats on 2nd day of testing the EPM.
The t-test indicates a significant difference between groups (** $p < 0.01$) in the time spent in the open arms.

Table S13. Results for zone transition within the closed arms in the EPM following RMg electrical stimulation (RMg-ST) or a naïve procedure in control rats (RMg-Sham) – corresponding to Figure 4A in the manuscript.

[n – number of individuals per group, K-S states from Kolmogorov-Smirnov normality test (L – Liliefors significant level); SD states from the standard deviation; SE states from the standard error; IQR states from the interquartile range; t-test states from Student's t-test; U states from Mann-Whitney U test]

zone transition within the closed arms	day	group	n	mean	median	K-S	SD	SE	IQR	t-test	U
	1	RMg-Sham	14	5.1	5	D = 0.197<br>p > 0.20	1.64	0.44	1.8	**t = -3.558<br>p < 0.001**	**Z = -3.320<br>p < 0.001**
						L < 0.15				df 33	U = 48.5
		RMg-ST	21	10.1	10	D = 0.144<br>p > 0.20	5.09	1.11	6.0		
						L > 0.20					
	2	RMg-Sham	14	7.3	7	D = 0.181<br>p > 0.20	2.84	0.76	2.2	**t = -3.321<br>p < 0.01**	**Z = -3.384<br>p < 0.001**
						L > 0.20				df 33	U = 46.0
		RMg-ST	21	13.2	12	D = 0.177<br>p > 0.20	6.24	1.36	6.0		
						L < 0.10					

Table S14. Results of the paired-samples t-test comparing consecutive days for zone transitions within the closed arms in the EPM.

RMg-Sham	**t = -4.016**<br>**p < 0.01**		RMg-ST	**t = -6.398**<br>**p < 0.000**
	df 13			df 20

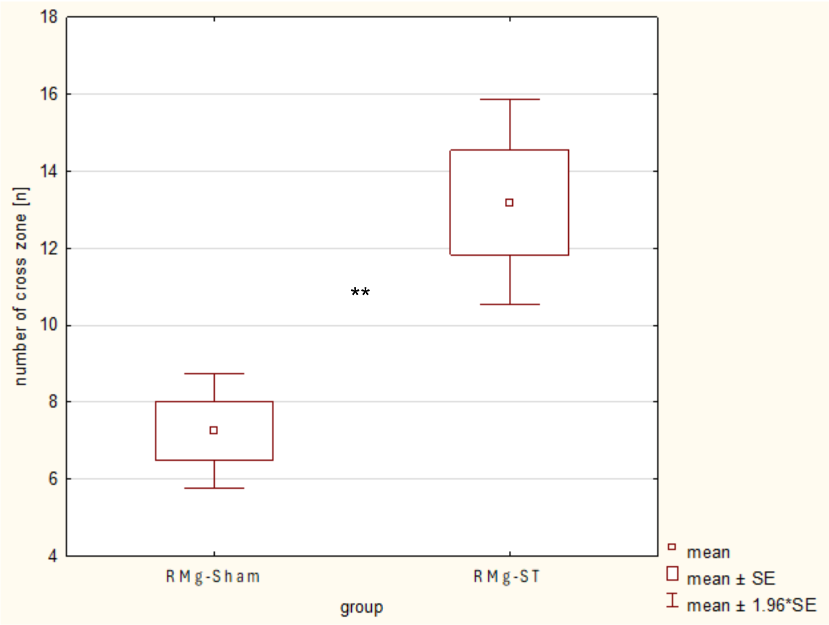

Figure S6. Comparison of zone transition within the closed arms on 2nd day of testing in the EPM.
The t-test indicates a significant difference between groups (** $p < 0.01$) in the number of zone transitions within the closed arms.

Table S15. Results for the number of entries into the open arms in the EPM following RMg electrical stimulation (RMg-ST) or a naïve procedure in control rats (RMg-Sham) – corresponding to Figure 4B in the manuscript.

[n – number of individuals per group, K-S states from Kolmogorov-Smirnov normality test (L – Liliefors significant level); SD states from the standard deviation; SE states from the standard error; IQR states from the interquartile range; t-test states from Student's t-test; U states from Mann-Whitney U test]

number of entries into the open arms	day	group	n	mean	median	K-S	SD	SE	IQR	t-test	U
	1	RMg-Sham	14	2.0	2	D = 0.214<br>p > 0.20	1.75	0.47	1.8	**t = -3.296<br>p < 0.01**	**Z = -3.377<br>p < 0.001**
						L < 0.10				df 33	U = 47.5
		RMg-ST	21	4.0	4	D = 0.206<br>p > 0.20	1.83	0.40	2.0		
						L < 0.01					
	2	RMg-Sham	14	2.6	3	D = 0.272<br>p > 0.20	1.99	0.53	1.8	**t = -4.228<br>p < 0.000**	**Z = -3.779<br>p < 0.000**
						L < 0.01				df 33	U = 36.0
		RMg-ST	21	5.4	5	D = 0.277<br>p < 0.05	1.88	0.41	1.0		
						L < 0.01					

Table S16. Results of the Wilcoxon singed-rank test comparing consecutive days for the number of entries into the open arms in the EPM.

RMg-Sham	**Z = 2.366<br>p < 0.05**
	N 7

RMg-ST	**t = 3.920<br>p < 0.000**
	N 20

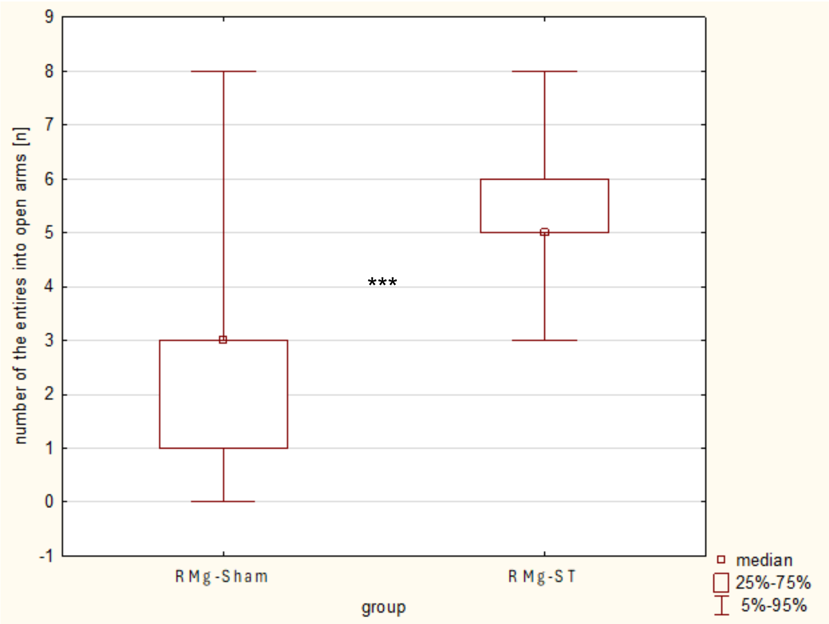

Figure S7. Comparison of the number of entries into the open arms on 2nd day of testing in the EPM.
The Mann–Whitney U test indicates a significant difference between groups (*** $p < 0.001$) for the number of entries into the open arms.

Table S17. Results for the time spent in the open arms in the EPM following RMg electrical stimulation (RMg-ST) or a naïve procedure in control rats (RMg-Sham) – corresponding to Figure 5A in the manuscript.

[n – number of individuals per group, K-S states from Kolmogorov-Smirnov normality test (L – Liliefors significant level); SD states from the standard deviation; SE states from the standard error; IQR states from the interquartile range; t-test states from Student's t-test; U states from Mann-Whitney U test]

time spent in the open arms

day	group	n	mean	median	K-S	SD	SE	IQR	t-test	U
1	RMg-Sham	14	6.5	6	D = 0.210 p > 0.20	6.09	1.63	5.5	**t = -3.353 p < 0.01**	**Z = -3.165 p < 0.01**
					L < 0.10				df 33	U = 52.5
	RMg-ST	21	15.2	14	D = 0.159 p > 0.20	8.33	1.82	4.8		
					L < 0.15					
2	RMg-Sham	14	8.8	9	D = 0.182 p > 0.20	7.66	2.05	8.0	**t = -3.627 p < 0.001**	**Z = -3.165 p < 0.05**
					L > 0.20				df 33	U = 52.5
	RMg-ST	21	20.2	22	D = 0.124 p > 0.20	9.91	2.16	8.9		
					L > 0.20					

Table S18. Results of the paired-samples t-test comparing consecutive days for the time spent in the open arms in the EPM.

RMg-Sham	**t = -4.383<br>p < 0.001**
	df 13

RMg-ST	**t = -6.677<br>p < 0.000**
	df 20

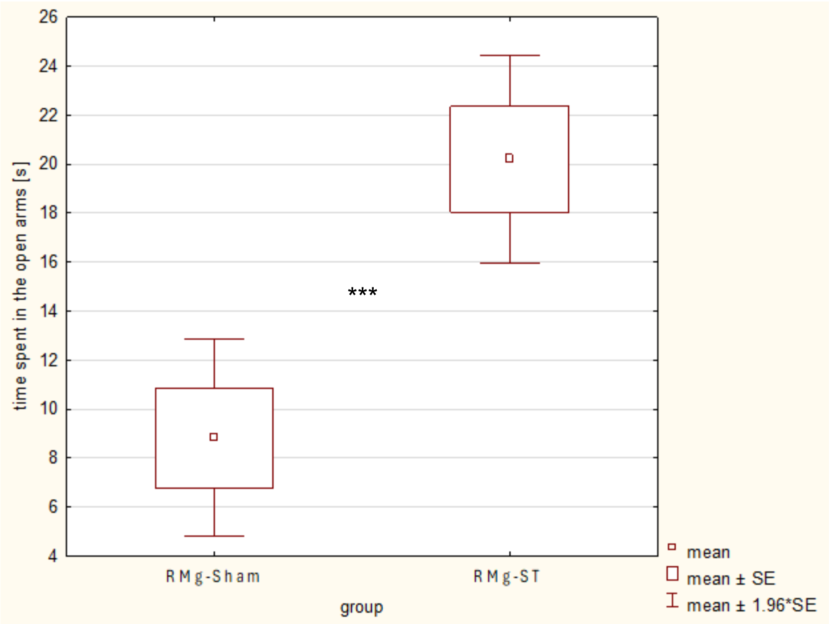

Figure S8. Comparison of the time spent in the open arms on 2nd day of testing in the EPM.
The t-test indicates a significant difference between groups (*** $p < 0.001$) in the time spent in the open arms.

Table S19. Results for the latency to the first movement of rats in the EPM test following RMg electrical stimulation (RMg-ST) or a naïve procedure in control rats (RMg-Sham)– corresponding to Figure 5B in the manuscript.

[n – number of individuals per group, K-S states from Kolmogorov-Smirnov normality test (L – Liliefors significant level); SD states from the standard deviation; SE states from the standard error; IQR states from the interquartile range; t-test states from Student's t-test; U states from Mann-Whitney U test]

**latency to the first movement**	**day**	**group**	**n**	**mean**	**median**	**K-S**	**SD**	**SE**	**IQR**	**t-test**	**U**
	1	RMg-Sham	14	42.5	42	D = 0.179<br>p > 0.20	43.29	11.57	45.7	**t = 3.299<br>p < 0.01**	**Z = 2.155<br>p < 0.05**
						L > 0.20				df 33	U = 85.0
		RMg-ST	21	10.8	9	D = 0.116<br>p > 0.20	8.02	1.75	9.7		
						L > 0.20					
	2	RMg-Sham	14	32.7	27	D = 0.224<br>p > 0.20	36.72	9.81	34.5	**t =3.073<br>p < 0.01**	**Z = 2.071<br>p < 0.05**
						L < 0.10				df 33	U = 82.5
		RMg-ST	21	7.8	6	D = 0.159<br>p > 0.20	5.81	1.27	7.9		
						L < 0.15					

Table S20. Results of the paired-samples t-test comparing consecutive days for the latency to the first movement in the EPM.

RMg-Sham	t = 4.152<br>p < 0.001	RMg-ST	t = 4.952<br>p < 0.000
	df 13		df 20

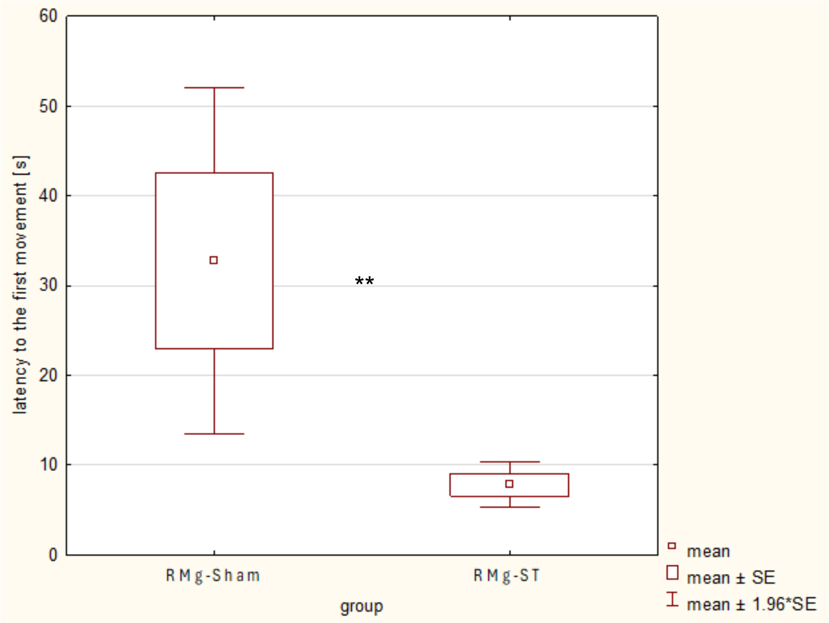

Figure S9. Comparison of the latency to the first movement of rats on 2nd day of testing in the EPM.
The t-test indicates a significant difference between groups (** $p < 0.01$) in the time spent in the open arms.

Table S21. Results of the two-way ANOVA, including tests of significance, effect sizes, and statistical power, for the latency to the first movement of rats in the EPM.

Effects	SS	degrees of freedom	MS	F	p
Intercept	3.4E+05	1	2.0E+09	478.494	0.00
group	39.3	1	39.3	0.055	0.81
distance	1.3E+05	1	1.3E+05	187.158	0.00
group × distance	1.5E+04	1	1.5E+04	21.594	0.00
Error	4.7E+04	66	7.1E+02		

Table S22. Results of Levene's test for homogeneity of variance for the group × distance effects on the latency to the first movement of rats in the EPM.

Effects	SS	MS	F	p
Intercept	1.6E+03	3.2E+02	4.842	0.01

Table S23. Games–Howell post-hoc analysis of the group × distance effects for the latency to the first movement of rats in the EPM.

group		{1} 101.19	{2} 42.49	{3} 129.86	{4} 10.76
RMg-Sham	distance in the maze		0.00	0.01	0.00
RMg-Sham	latency to the first movement	0.00		0.00	0.01
RMg-ST	distance in the maze	0.01	0.00		0.00
RMg-ST	latency to the first movement	0.00	0.01	0.00	

Table S24. Results of the two-way ANOVA, including tests of significance, effect sizes, and statistical power, for the time spent in the open arms in the EPM.

Effects	SS	degrees of freedom	MS	F	p
Intercept	2.8E+05	1	2.8E+05	784.152	0.00
group	6.7E+03	1	6.7E+03	18.592	0.00
distance	1.7E+05	1	1.7E+05	473.231	0.00
group × distance	1.3E+03	1	1.3E+03	3.467	0.07
Error	2.4E+04	66	3.6E+02		

Table S25. Results for the latency to reach the platform during the pretest in the MWM following RMg electrical stimulation (RMg-ST) or a naïve procedure in control rats (RMg-Sham).

[n – number of individuals per group, K-S states from Kolmogorov-Smirnov normality test (L – Liliefors significant level); SD states from the standard deviation; SE states from the standard error; IQR states from the interquartile range; t-test states from Student's t-test; U states from Mann-Whitney U test]

PRETEST
latency

**group**	**n**	**mean**	**median**	**K-S**	**SD**	**SE**	**IQR**
RMg-Sham	28	37.6	38	D = 0.177<br>p > 0.20	18.03	3.41	12.3
				L > 0.05			
RMg-ST	42	33.2	12	D = 0.134<br>p > 0.20	12.13	1.87	11.8
				L < 0.10			

**t-test**	**U**
**t = 1.235**<br>**p = 0.22**	**Z = 1.822**<br>**p = 0.07**
df 68	U = 435.5

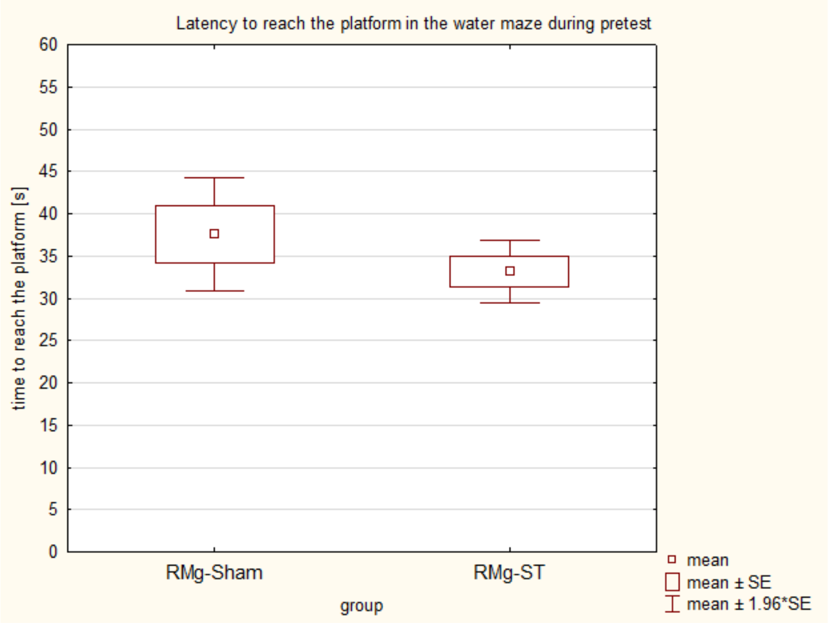

Figure S10. Comparison of the latency to reach the platform during the pretest phase in the MWM.

Table S26. Results for the distance swam during the pretest in the MWM following RMg electrical stimulation (RMg-ST) or a naïve procedure in control rats (RMg-Sham).

[n – number of individuals per group, K-S states from Kolmogorov-Smirnov normality test (L – Liliefors significant level); SD states from the standard deviation; SE states from the standard error; IQR states from the interquartile range; t-test states from Student's t-test; U states from Mann-Whitney U test]

PRETEST
distance

**group**	**n**	**mean**	**median**	**K-S**	**SD**	**SE**	**IQR**
RMg-Sham	28	1010.6	919	D = 0.166<br>p > 0.20	503.21	95.10	870.3
				L > 0.05			
RMg-ST	42	762.9	781	D = 0.076<br>p > 0.20	272.58	42.06	311.4
				L > 0.20			

**t-test**	**U**
**t = 2.664<br>p = 0.01**	**Z = -1.696<br>p = 0.05**
df 68	U = 446.0

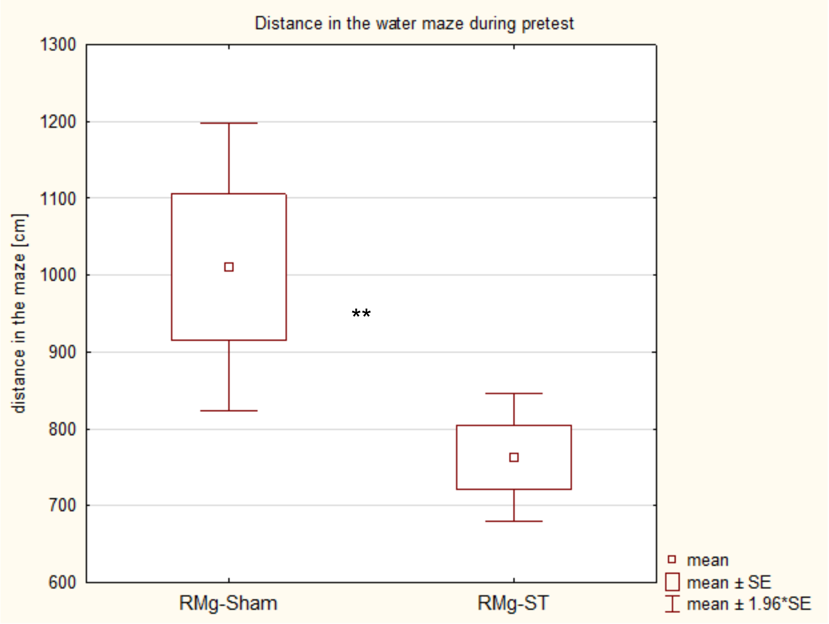

Figure S11. Comparison of the distance swam in the maze during the spatial memory test phase in the MWM. The t-test indicates a significant difference between groups (** p < 0.01) for the distance swam in the maze.

Table S27. Results for the latency to reach the platform on 1st day of the spatial memory test in the MWM following RMg electrical stimulation (RMg-ST) or a naïve procedure in control rats (RMg-Sham) – corresponding to Figure 6 in the manuscript.

[n – number of individuals per group, K-S states from Kolmogorov-Smirnov normality test (L – Liliefors significant level); SD states from the standard deviation; SE states from the standard error; IQR states from the interquartile range; t-test states from Student's t-test; U states from Mann-Whitney U test]

TEST – 1st day
latency

trial	group	n	mean	median	K-S	SD	SE	IQR	t-test	U
1	RMg-Sham	14	44.7	49	D = 0.150<br>p > 0.20	20.45	5.47	29.8	**t = 5.669<br>p < 0.000**	**Z = 3.822<br>p < 0.000**
					L > 0.20				df 33	U = 33.0
	RMg-ST	21	16.9	17	D = 0.123<br>p > 0.20	7.81	1.70	13.4		
					L > 0.10					
2	RMg-Sham	14	29.0	26	D = 0.179<br>p > 0.20	12.88	3.44	21.1	**t = 5.320<br>p < 0.000**	**Z = 3.956<br>p < 0.000**
					L > 0.20				df 33	U = 29.0
	RMg-ST	21	10.9	10	D = 0.130<br>p > 0.20	7.18	1.57	9.1		
					L > 0.20					
3	RMg-Sham	14	17.6	17	D = 0.181<br>p > 0.20	7.12	1.90	8.3	**t = 3.681<br>p < 0.000**	**Z = 3.317<br>p < 0.001**
					L > 0.20				df 33	U = 48.0
	RMg-ST	21	9.2	6	D = 0.213<br>p < 0.20	6.24	1.36	9.4		
					L < 0.01					
4	RMg-Sham	14	10.4	10	D = 0.141<br>p > 0.20	4.27	1.14	5.4	**t = 3.415<br>p = 0.00**	**Z = 3.148<br>p < 0.01**
					L > 0.20				df 33	U = 53.0
	RMg-ST	21	5.2	3	D = 0.250<br>p < 0.10	4.50	0.98	3.3		
					L < 0.01					

Table S28. Results for the latency to reach the platform on 2nd day of the spatial memory test in the MWM following RMg electrical stimulation (RMg-ST) or a naïve procedure in control rats (RMg-Sham) – corresponding to Figure 6 in the manuscript.
[n – number of individuals per group, K-S states from Kolmogorov-Smirnov normality test (L – Liliefors significant level); SD states from the standard deviation; SE states from the standard error; IQR states from the interquartile range; t-test states from Student's t-test; U states from Mann-Whitney U test]

TEST – 2nd day
latency

trial	group	n	mean	median	K-S	SD	SE	IQR	t-test	U
1	RMg-Sham	14	45.3	50	$D = 0.157$<br>$p > 0.20$	21.97	5.87	32.2	**$t = 5.582$<br>$p < 0.000$**	**$Z = 3.722$<br>$p < 0.000$**
					$L > 0.20$				df 33	$U = 36.0$
	RMg-ST	21	16.3	15	$D = 0.102$<br>$p > 0.20$	7.79	1.70	11.8		
					$L > 0.20$					
2	RMg-Sham	14	29.0	26	$D = 0.135$<br>$p > 0.20$	12.80	3.42	16.3	**$t = 5.540$<br>$p < 0.000$**	**$Z = 4.192$<br>$p < 0.000$**
					$L > 0.20$				df 33	$U = 22.0$
	RMg-ST	21	10.5	10	$D = 0.129$<br>$p > 0.20$	6.95	1.52	8.4		
					$L > 0.20$					
3	RMg-Sham	14	17.8	17	$D = 0.122$<br>$p > 0.20$	7.51	2.01	9.7	**$t = 4.009$<br>$p < 0.000$**	**$Z = 3.335$<br>$p < 0.001$**
					$L > 0.20$				df 33	$U = 47.5$
	RMg-ST	21	8.8	6	$D = 0.215$<br>$p < 0.20$	5.73	1.25	9.9		
					$L < 0.01$					
4	RMg-Sham	14	10.3	10	$D = 0.159$<br>$p > 0.20$	4.11	1.10	5.6	**$t = 3.913$<br>$p < 0.000$**	**$Z = 3.521$<br>$p < 0.000$**
					$L > 0.20$				df 33	$U = 53.0$
	RMg-ST	21	4.8	3.1	$D = 0.256$<br>$p < 0.10$	4.05	0.88	3.7		
					$L < 0.01$					

Table S29. Results for the distance swam in the maze on 1st day of the spatial memory test in the MWM following RMg electrical stimulation (RMg-ST) or a naïve procedure in control rats (RMg-Sham)– corresponding to Figure 7 in the manuscript.

[n – number of individuals per group, K-S states from Kolmogorov-Smirnov normality test (L – Liliefors significant level); SD states from the standard deviation; SE states from the standard error; IQR states from the interquartile range; t-test states from Student's t-test; U states from Mann-Whitney U test]

TEST – 1st day
distance

trial	group	n	mean	median	K-S	SD	SE	IQR	t-test	U
1	RMg-Sham	14	1484.1	1487	D = 0.133<br>p > 0.20	638.17	170.56	778.4	**t = 4.287<br>p < 0.000**	**Z = 3.721<br>p < 0.000**
					L > 0.20				df 33	U = 36.0
	RMg-ST	21	630.2	564	D = 0.221<br>p > 0.15	534.13	115.56	419.6		
					L < 0.01					
2	RMg-Sham	14	765.8	759	D = 0.102<br>p > 0.20	248.40	66.39	393.8	**t = 6.746<br>p < 0.000**	**Z = 4.462<br>p < 0.000**
					L > 0.20				df 33	U = 14.0
	RMg-ST	21	297.4	292	D = 0.120<br>p > 0.20	163.45	35.67	236.7		
					L > 0.20					
3	RMg-Sham	14	725.0	502	D = 0.138<br>p > 0.20	256.25	68.48	260.4	**t = 4.504<br>p < 0.000**	**Z = 3.721<br>p < 0.000**
					L > 0.20				df 33	U = 36.0
	RMg-ST	21	210.5	158	D = 0.209<br>p < 0.20	144.73	31.58	170.1		
					L < 0.01					
4	RMg-Sham	14	315.4	352	D = 0.216<br>p > 0.20	97.38	26.03	146.8	**t = 4.770<br>p < 0.00**	**Z = 3.687<br>p < 0.000**
					L < 0.10				df 33	U = 37.0
	RMg-ST	21	146.1	97	D = 0.213<br>p < 0.20	106.27	23.19	136.6		
					L < 0.01					

Table S30. Results for the distance swam in the maze on 2nd day of the spatial memory test in the MWM following RMg electrical stimulation (RMg-ST) or a naïve procedure in control rats (RMg-Sham)– corresponding to Figure 7 in the manuscript.

[n – number of individuals per group, K-S states from Kolmogorov-Smirnov normality test (L – Liliefors significant level); SD states from the standard deviation; SE states from the standard error; IQR states from the interquartile range; t-test states from Student's t-test; U states from Mann-Whitney U test]

TEST – 2nd day
distance

**trial**	**group**	**n**	**mean**	**median**	**K-S**	**SD**	**SE**	**IQR**	**t-test**	**U**
1	RMg-Sham	14	1401.8	1444	D = 0.123 p > 0.20	581.62	155.44	793.9	**t = 4.287 p < 0.000**	**Z = 3.687 p < 0.000**
					L > 0.20				df 33	U = 37.0
	RMg-ST	21	618.3	553	D = 0.260 p > 0.10	560.38	122.28	385.6		
					L < 0.01					
2	RMg-Sham	14	725.0	700	D = 0.135 p > 0.20	237.67	63.52	381.3	**t = 6.746 p < 0.000**	**Z = 4.462 p < 0.000**
					L > 0.20				df 33	U = 14.0
	RMg-ST	21	280.6	280	D = 0.114 p > 0.20	152.13	33.20	215.5		
					L > 0.20					
3	RMg-Sham	14	489.8	461	D = 0.147 p > 0.20	248.88	66.52	259.4	**t = 4.504 p < 0.000**	**Z = 3.552 p < 0.000**
					L > 0.20				df 33	U = 41.0
	RMg-ST	21	210.3	158	D = 0.208 p < 0.20	144.84	31.61	172.7		
					L < 0.01					
4	RMg-Sham	14	298.1	328	D = 0.181 p > 0.20	91.54	24.47	150.1	**t = 4.770 p < 0.00**	**Z = 3.552 p < 0.000**
					L > 0.10				df 33	U = 41.0
	RMg-ST	21	145.9	97	D = 0.212 p < 0.20	106.34	23.20	136.6		
					L < 0.01					

Table S31. Results of the paired-samples t-test or Wilcoxon signed-rank test comparing the latency to reach the platform between consecutive days in the MWM.

trial 1	RMg-Sham	**t = -0.689 p = 0.50**	RMg-ST	**t = 3.420 p < 0.01**	
		df 13		df 20	
trial 2	RMg-Sham	**t = -0.003 p = 0.98**	RMg-ST	**t = 3.020 p < 0.01**	
		df 13		df 20	
trial 3	RMg-Sham	**t = -0.438 p = 0.69**	RMg-ST	**t = 2.507 p < 0.05**	**Z = 2.728 p = 0.006**
		df 13		df 20	T = 37.0
trial 4	RMg-Sham	**t = -0.139 p = 0.89**	RMg-ST	**t = 2.972 p < 0.01**	**Z = 2.937 p = 0.003**
		df 13		df 20	T = 31.0

Table S32. Results of the paired-samples t-test or Wilcoxon signed-rank test comparing the distance swam in the maze between consecutive days in the MWM.

trial 1	RMg-Sham	**t = 3.685 p < 0.01**	df 13	RMg-ST	**t = 0.994 p = 0.33**	df 20	**Z = 1.825 p = 0.07**	T = 63.0
trial 2	RMg-Sham	**t = 5.137 p < 0.001**	df 13	RMg-ST	**t = 4.717 p < 0.001**	df 20		
trial 3	RMg-Sham	**t = 5.088 p < 0.000**	df 13	RMg-ST	**t = 1.000 p = 0.33**	df 20	**Z = 2.728 p = 0.006**	T = 37.0
trial 4	RMg-Sham	**t = 4.489 p < 0.000**	df 13	RMg-ST	**t = 1.000 p < 0.33**	df 20	**Z = 2.937 p = 0.003**	T = 31.0

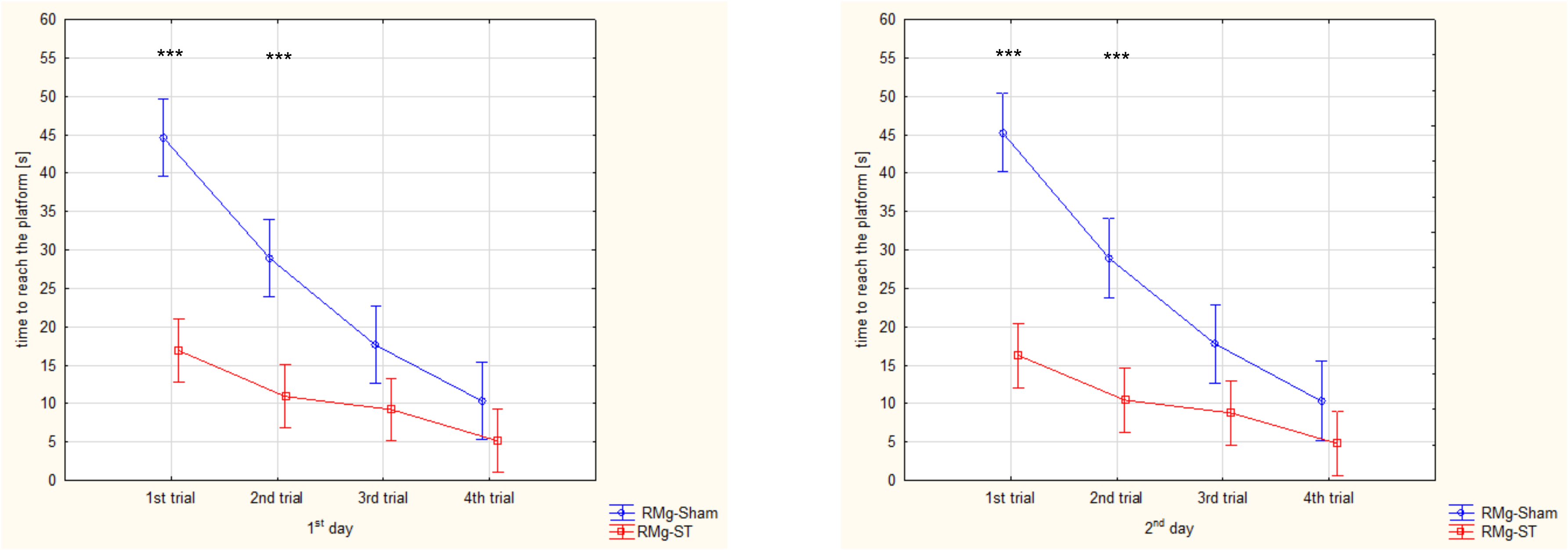

Figure S12. Comparison of the latency to reach the platform during the test phase in the MWM.
A two-way ANOVA indicates a significant interaction between stimulation group and trial number ($F_{(7, 264)} = 8.391$, $p = 0.000$; *** $p < 0.001$).

Table S33. Results of the two-way ANOVA, including univariate tests of significance, effect sizes, and statistical power for the latency to reach the platform in the MWM.

Effects	SS	degrees of freedom	MS	F	p
Intercept	8.4E+04	1	8.6E+04	930.956	0.00
group	1.5E+04	1	1.5E+04	167.121	0.00
trial	2.0E+04	7	2.8E+03	30.614	0.00
group × trial	5.4E+03	7	7.8E+02	8.391	0.00
Error	2.4E+04	264	9.3E+01		

Table S34. Results of Levene's test for the homogeneity of variance for the group × trial effects on the latency to reach the platform in the MWM.

Effects	SS	MS	F	p
Intercept	3.0E+02	2.8E+01	10.742	0.00

Table S35. Games–Howell post-hoc analysis of the group × trail effects for the latency to reach the platform in the MWM.

group		{1}<br>44.7	{2}<br>45.3	{3}<br>28.9	{4}<br>29.0	{5}<br>17.6	{6}<br>17.8	{7}<br>10.4	{8}<br>10.3	{9}<br>16.9	{10}<br>16.3	{11}<br>10.9	{12}<br>10.5	{13}<br>9.2	{14}<br>8.8	{15}<br>5.2	{16}<br>4.8
RMg-Sham	Trial 1 1st day		0.86	0.01	0.00	0.00	0.00	0.00	0.00	0.00	0.00	0.00	0.00	0.00	0.00	0.00	0.00
RMg-Sham	Trial 1 2nd day	0.86		0.00	0.01	0.00	0.00	0.00	0.00	0.00	0.00	0.00	0.00	0.00	0.00	0.00	0.00
RMg-Sham	Trial 2 1st day	0.00	0.00		0.95	0.00	0.00	0.00	0.00	0.00	0.00	0.00	0.00	0.00	0.00	0.00	0.00
RMg-Sham	Trial 2 2nd day	0.00	0.00	0.95		0.00	0.00	0.00	0.00	0.00	0.00	0.00	0.00	0.00	0.00	0.00	0.00
RMg-Sham	Trial 3 1st day	0.00	0.00	0.00	0.00		0.96	0.24	0.30	0.83	0.91	0.18	0.20	0.18	0.16	0.01	0.01
RMg-Sham	Trial 3 2nd day	0.00	0.00	0.00	0.00	0.96		0.28	0.33	0.96	0.97	0.24	0.24	0.20	0.17	0.01	0.01
RMg-Sham	Trial 4 1st day	0.00	0.00	0.00	0.00	0.24	0.28		0.99	0.28	0.28	0.98	0.97	0.94	0.97	0.52	0.56
RMg-Sham	Trial 4 2nd day	0.00	0.00	0.00	0.00	0.30	0.33	0.99		0.35	0.38	0.98	0.98	0.74	0.89	0.40	0.46
RMg-ST	Trial 1 1st day	0.00	0.01	0.00	0.00	0.83	0.96	0.28	0.35		0.85	0.17	0.21	0.24	0.22	0.01	0.01
RMg-ST	Trial 1 2nd day	0.00	0.00	0.00	0.00	0.91	0.97	0.28	0.38	0.85		0.11	0.19	0.28	0.27	0.02	0.02
RMg-ST	Trial 2 1st day	0.00	0.00	0.00	0.00	0.18	0.24	0.98	0.98	0.17	0.11		0.88	0.98	0.98	0.59	0.59
RMg-ST	Trial 2 2nd day	0.00	0.00	0.00	0.00	0.20	0.24	0.97	0.98	0.21	0.19	0.88		0.98	0.98	0.60	0.62
RMg-ST	Trial 3 1st day	0.00	0.00	0.00	0.00	0.18	0.20	0.94	0.74	0.24	0.28	0.98	0.98		0.90	0.44	0.55
RMg-ST	Trial 3 2nd day	0.00	0.00	0.00	0.00	0.16	0.17	0.97	0.89	0.22	0.27	0.98	0.98	0.90		0.27	0.46
RMg-ST	Trial 4 1st day	0.00	0.00	0.00	0.00	0.01	0.01	0.52	0.40	0.01	0.02	0.59	0.60	0.44	0.27		0.92
RMg-ST	Trial 4 2nd day	0.00	0.00	0.00	0.00	0.01	0.01	0.56	0.46	0.01	0.02	0.59	0.62	0.55	0.46	0.92	

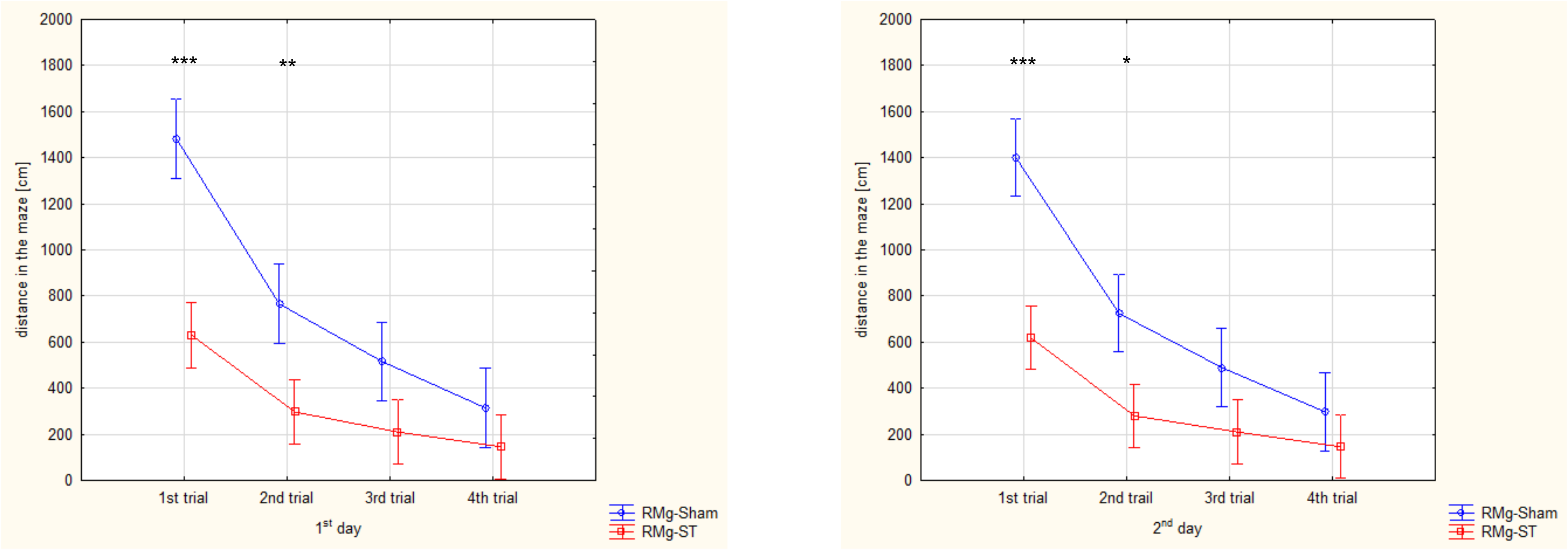

Figure S13. Comparison of the distance swam in the maze during the test phase in the MWM.
A two-way ANOVA indicates a significant interaction between stimulation group and trial number ($F_{(7, 264)} = 5.664$, $p = 0.000$; * $p < 0.05$; ** $p < 0.01$; *** $p < 0.001$).

Table S36. Results of the one-way ANOVA, including univariate tests of significance, effect sizes, and statistical power for the distance swam in the maze in the MWM.

Effects	SS	degrees of freedom	MS	F	p
Intercept	7.6E+07	1	7.6E+07	737.119	0.00
group	1.3E+07	1	1.3E+07	120.894	0.00
trial	2.5E+07	7	3.6E+06	34.862	0.00
group × trial	4.1E+06	7	5.9E+05	5.664	0.00
Error	3.7E+07	264	1.0E+05		

Table S37. Results of Levene's test for the homogeneity of variance for group × trial effects on the distance swam in the maze in the MWM.

Effects	SS	MS	F	p
Intercept	3.0E+02	2.8E+01	5.571	0.00

Table S38. Games–Howell post-hoc analysis of the group × trail effects on the distance swam in the maze in the MWM.

group	trail	{1} 1484.1	{2} 1401.8	{3} 765.8	{4} 725.0	{5} 515.7	{6} 489.8	{7} 315.4	{8} 298.1	{9} 630.2	{10} 618.3	{11} 297.6	{12} 280.6	{13} 210.5	{14} 210.3	{15} 146.1	{16} 4.8
RMg-Sham	Trial 1 1st day		0.98	0.00	0.00	0.00	0.00	0.00	0.00	0.00	0.00	0.00	0.00	0.00	0.00	0.00	0.00
RMg-Sham	Trial 1 2nd day	0.98		0.00	0.00	0.00	0.00	0.00	0.00	0.00	0.00	0.00	0.00	0.00	0.00	0.00	0.00
RMg-Sham	Trial 2 1st day	0.00	0.00		0.98	0.79	0.65	0.02	0.01	0.98	0.98	0.01	0.01	0.00	0.00	0.00	0.00
RMg-Sham	Trial 2 2nd day	0.00	0.00	0.98		0.94	0.86	0.06	0.04	0.98	0.98	0.04	0.02	0.00	0.00	0.00	0.00
RMg-Sham	Trial 3 1st day	0.00	0.00	0.79	0.94		0.98	0.96	0.92	0.98	0.98	0.92	0.86	0.47	0.47	0.15	0.15
RMg-Sham	Trial 3 2nd day	0.00	0.00	0.65	0.86	0.98		0.99	0.97	0.98	0.98	0.97	0.94	0.63	0.63	0.26	0.25
RMg-Sham	Trial 4 1st day	0.00	0.00	0.02	0.06	0.96	0.98		0.98	0.41	0.48	0.98	0.98	0.98	0.98	0.98	0.98
RMg-Sham	Trial 4 2nd day	0.00	0.00	0.01	0.04	0.92	0.97	0.98		0.31	0.38	0.98	0.98	0.98	0.98	0.98	0.98
RMg-ST	Trial 1 1st day	0.00	0.01	0.98	0.98	0.98	0.98	0.41	0.31		0.98	0.06	0.04	0.00	0.00	0.00	0.00
RMg-ST	Trial 1 2nd day	0.00	0.00	0.98	0.98	0.98	0.98	0.48	0.38	0.98		0.09	0.05	0.00	0.00	0.00	0.00
RMg-ST	Trial 2 1st day	0.00	0.00	0.01	0.04	0.92	0.97	0.98	0.98	0.06	0.09		0.98	0.98	0.98	0.98	0.98
RMg-ST	Trial 2 2nd day	0.00	0.00	0.00	0.02	0.86	0.94	0.98	0.98	0.04	0.05	0.98		0.98	0.98	0.99	0.98
RMg-ST	Trial 3 1st day	0.00	0.00	0.00	0.00	0.47	0.63	0.98	0.98	0.00	0.00	0.98	0.98		0.90	0.98	0.98
RMg-ST	Trial 3 2nd day	0.00	0.00	0.00	0.00	0.47	0.63	0.98	0.98	0.00	0.00	0.98	0.98	0.98		0.98	0.98
RMg-ST	Trial 4 1st day	0.00	0.00	0.00	0.00	0.15	0.26	0.98	0.98	0.00	0.00	0.98	0.98	0.98	0.98		0.98
RMg-ST	Trial 4 2nd day	0.00	0.00	0.00	0.00	0.15	0.25	0.98	0.98	0.00	0.00	0.98	0.98	0.98	0.98	0.98	

Table S39. Results of the probe phase in the water maze (MWM) test following RMg electrical stimulation (RMg-ST) or a naïve procedure in control rats (RMg-Sham) – corresponding to Figure 8 and 9 in the manuscript.

[n – number of individuals per group, K-S states from Kolmogorov-Smirnov normality test (L – Liliefors significant level); SD states from the standard deviation; SE states from the standard error; IQR states from the interquartile range; t-test states from Student's t-test; U states from Mann-Whitney U test]

parameter	group	n	mean	median	K-S	SD	SE	IQR	t-test	U
time spent in the critical quadrant	RMg-Sham	14	60.4	63	D = 0.139 p > 0.20	9.84	2.63	11.3	**t = 2.230 p = 0.03**	**Z = -1.751 p = 0.08**
					L > 0.20				df 33	U = 94.5
	RMg-ST	21	67.5	67	D = 0.096 p > 0.20	7.67	1.67	9.8		
					L > 0.20					
number of entrance to the critical quadrant	RMg-Sham	14	7.2	8	D = 0.258 p > 0.20	1.12	0.30	1.8	**t = -2.473 p = 0.02**	**Z = -2.239 p = 0.03**
					L < 0.10				df 33	U = 80.0
	RMg-ST	21	8.6	8	D = 0.146 p > 0.20	1.83	0.40	2.3		
					L > 0.20					
distance	RMg-Sham	14	2492.2	2511	D = 0.186 p > 0.20	297.76	79.58	231.9	**t = -2.763 p = 0.009**	**Z = -2.913 p = 0.004**
					L < 0.20				df 33	U = 60.0
	RMg-ST	21	2770.9	2778	D = 0.122 p > 0.20	288.66	62.99	277.8		
					L > 0.20					
total zone crossing	RMg-Sham	14	27.5	27	D = 0.130 p > 0.20	3.63	0.97	3.4	**t = -1.250 p = 0.22**	**Z = -1.414 p = 0.16**
					L > 0.20				df 33	U = 104.5
	RMg-ST	21	29.6	29	D = 0.113 p > 0.20	5.47	1.19	7.3		
					L > 0.20					

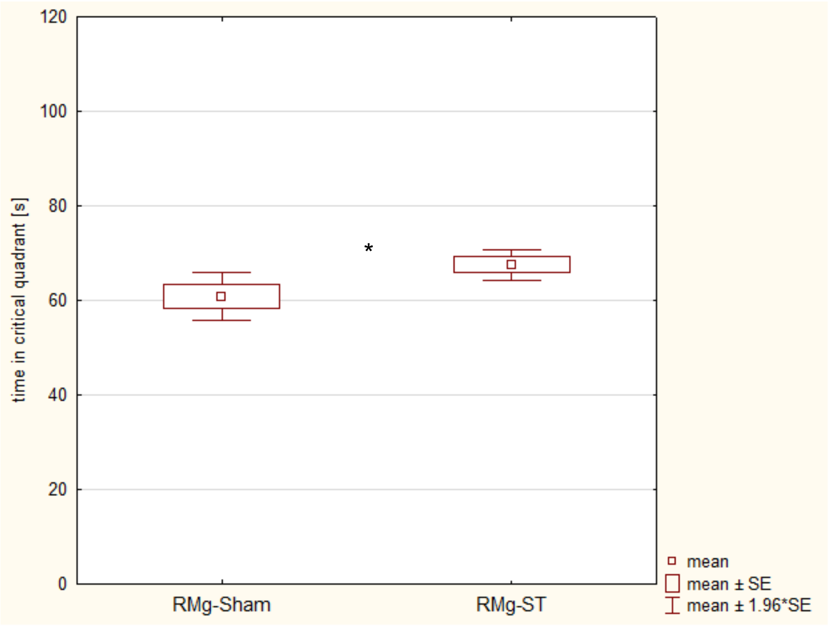

Figure S14. Comparison of the time spent in the critical quadrant (NE) during the probe phase in the MWM. The t-test indicates a significant difference between groups (* $p < 0.05$) for the time spent in the critical quadrant (NE).

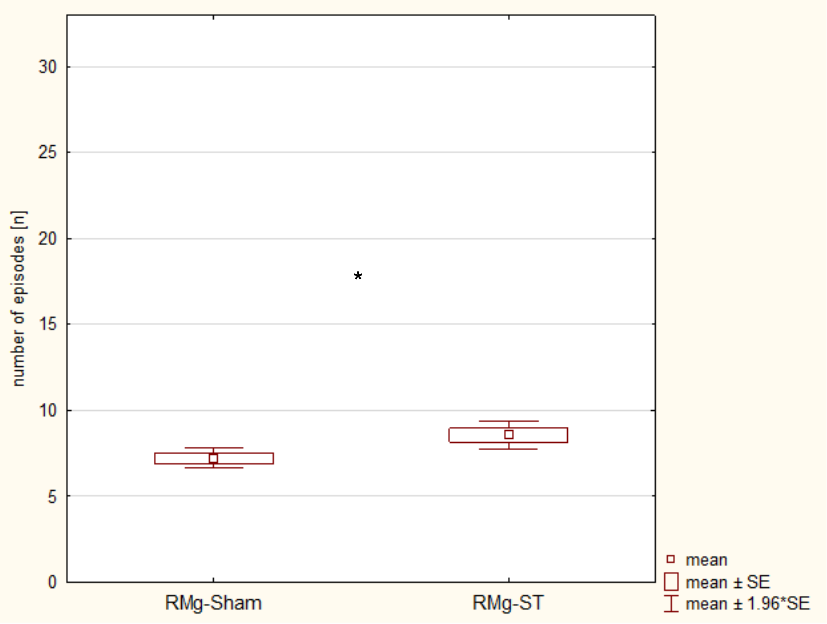

Figure S15. Comparison of entries into the critical quadrant (NE) during the probe phase in the MWM.
The t-test indicates a significant difference between groups (* $p < 0.05$) for entries into the critical quadrant (NE).

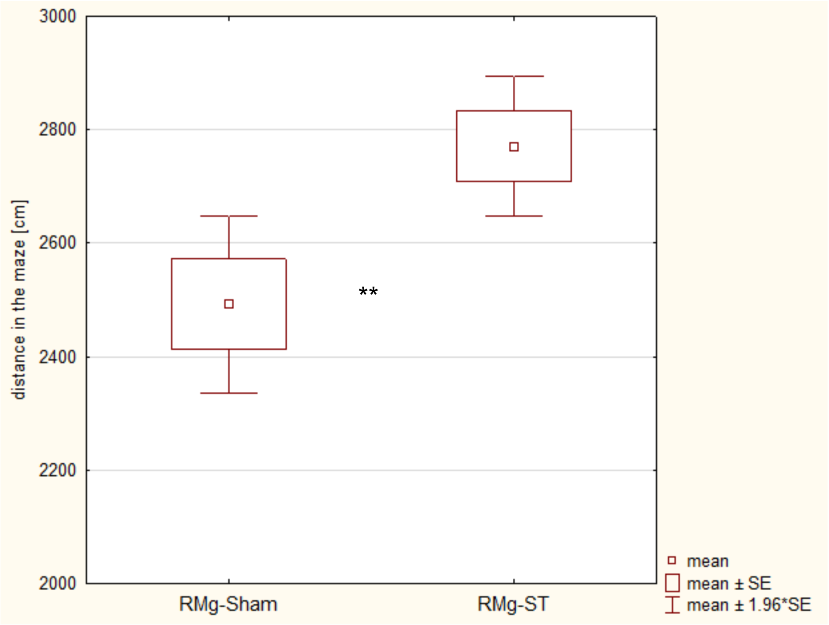

Figure S16. Comparison of the distance swam in the maze during the probe phase in the MWM.
The t-test indicates a significant difference between groups (** $p < 0.01$) for the distance swam in the maze.

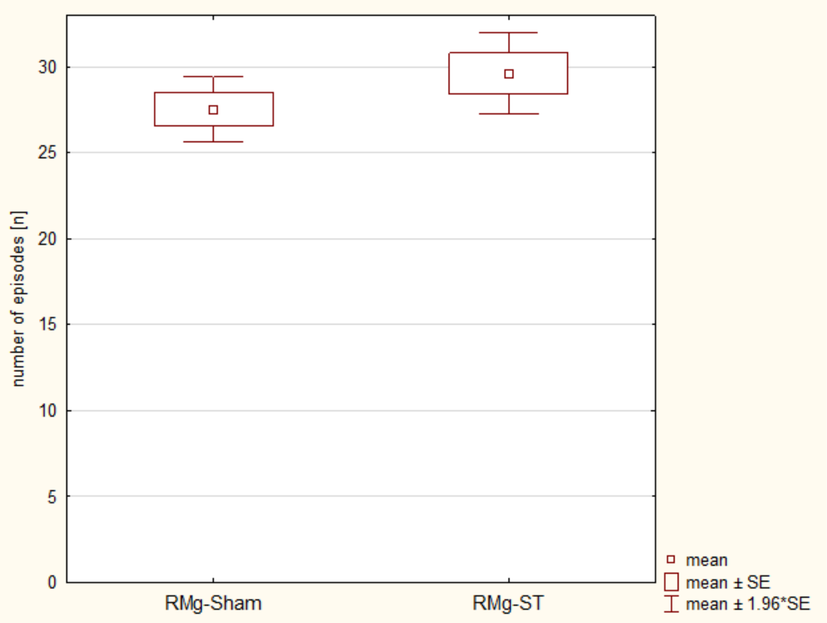

Figure S17. Comparison of entries into the critical quadrant (NE) and total zone crossings during the probe phase in the MWM.

Table S40. Results of double staining for the density of serotonergic (5-HT–positive; 5-HT+) cells and c-Fos–positive nuclei (c-Fos+) in the raphe magnus (RMg) following RMg electrical stimulation (RMg-ST) or a naïve procedure in control rats (RMg-Sham)– corresponding to Figure 10 and 11 in the manuscript.
[n – number of individuals per group, K-S states from Kolmogorov-Smirnov normality test (L – Liliefors significant level); SD states from the standard deviation; SE states from the standard error; IQR states from the interquartile range; t-test states from Student's t-test; U states from Mann-Whitney U test]

parameter	group	n	mean	median	K-S	SD	SE	IQR	t-test	U
density of 5-HT+ cells [number/mm^2]	RMg-Sham	82	24.9	24	D = 0.129 p > 0.15	7.42	0.82	8.2	**t = -25.717 p = 0.000**	**Z = -12.037 p = 0.000**
					L < 0.01				df 199	U = 0.0
	RMg-ST	119	313.4	301	D = 0.100 p > 0.20	101.32	9.29	126.7		
					L < 0.01					
density of c-Fos+ nuclei [number/mm^2]	RMg-Sham	82	25.4	25	D = 0.048 p > 0.20	8.50	0.94	11.3	**t = -42.448 p = 0.000**	**Z = -11.751 p = 0.080**
					L > 0.20				df 202	U = 80.0
	RMg-ST	122	200.8	197	D = 0.084 p > 0.20	36.73	3.33	38.2		
					L < 0.10					

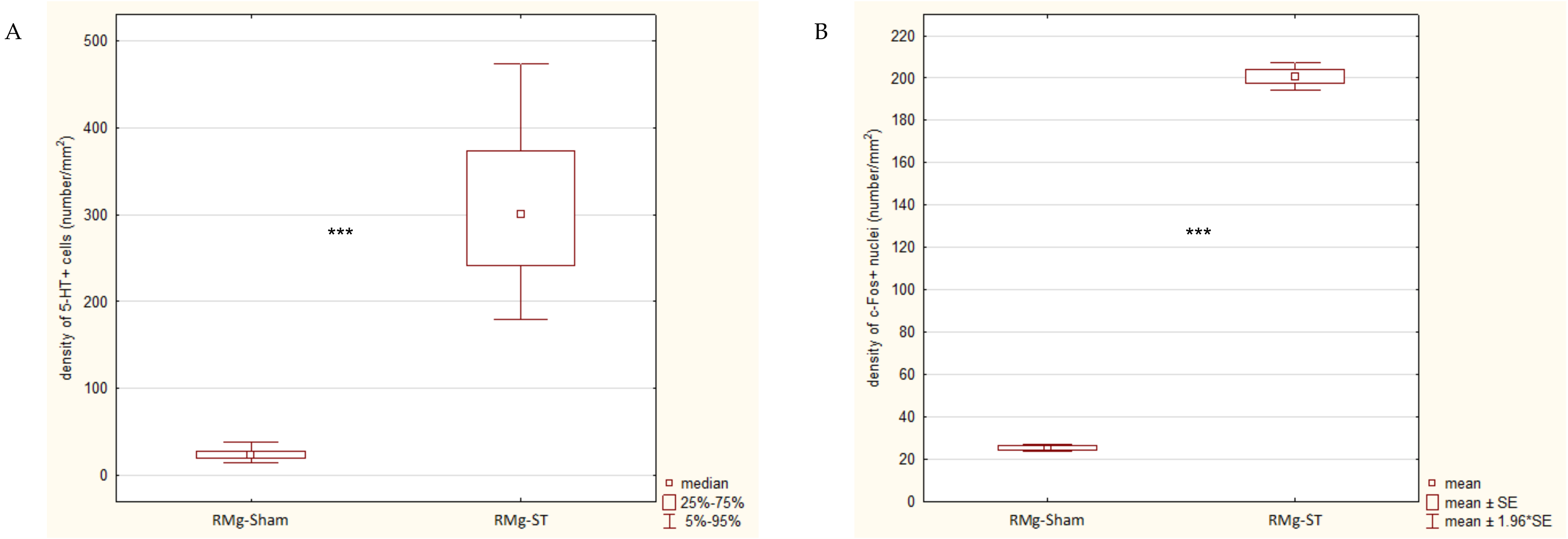

Figure S18. Comparison of the density of 5-HT–positive cells (5-HT+) (A) and the density of c-Fos–positive nuclei (c-Fos+) (B) in the raphe magnus following RMg electrical stimulation (RMg-ST) or a naïve procedure in control rats (RMg-Sham).
The Mann–Whitney U test indicates a significant difference between groups (*** $p < 0.001$) for 5-HT–positive cells.
The t-test indicates a significant difference between groups (*** $p < 0.001$) for c-Fos–positive nuclei.

Table S41. Results for the density of c-Fos–positive nuclei (c-Fos+) in selected brain structures following RMg electrical stimulation (RMg-ST) or a naïve procedure in control rats (RMg-Sham) — corresponding to Figure 12 to 15 in the manuscript.
[n – number of individuals per group, K-S states from Kolmogorov-Smirnov normality test (L – Liliefors significant level); SD states from the standard deviation; SE states from the standard error; IQR states from the interquartile range; t-test states from Student's t-test; U states from Mann-Whitney U test]

brain structure	group	n	mean	median	K-S	SD	SE	IQR	t-test	U
M2	RMg-Sham	136	11.7	11	D = 0.178<br>p < 0.01	4.78	0.41	4.1	**t = -25.054<br>p = 0.000**	**Z = -15.653<br>p = 0.000**
					L < 0.01				df 340	U = 0.0
	RMg-ST	206	117.3	108	D = 0.103<br>p < 0.05	48.99	3.41	80.0		
					L < 0.01					
ARC	RMg-Sham	67	24.4	23	D = 0.184<br>p < 0.05	12.59	1.54	11.1	**t = -17.760<br>p = 0.000**	**Z = -10.871<br>p = 0.000**
					L < 0.01				df 166	U = 27.0
	RMg-ST	101	128.6	133	D = 0.057<br>p > 0.20	46.87	4.66	67.0		
					L > 0.20					
DG	RMg-Sham	136	13.4	13	D = 0.095<br>p > 0.20	3.40	0.29	4.0	**t = -19.841<br>p = 0.000**	**Z = -13.374<br>p = 0.000**
					L > 0.05				df 342	U = 0.0
	RMg-ST	208	112.1	94	D = 0.134<br>p > 0.10	57.90	4.01	80.6		
					L > 0.05					

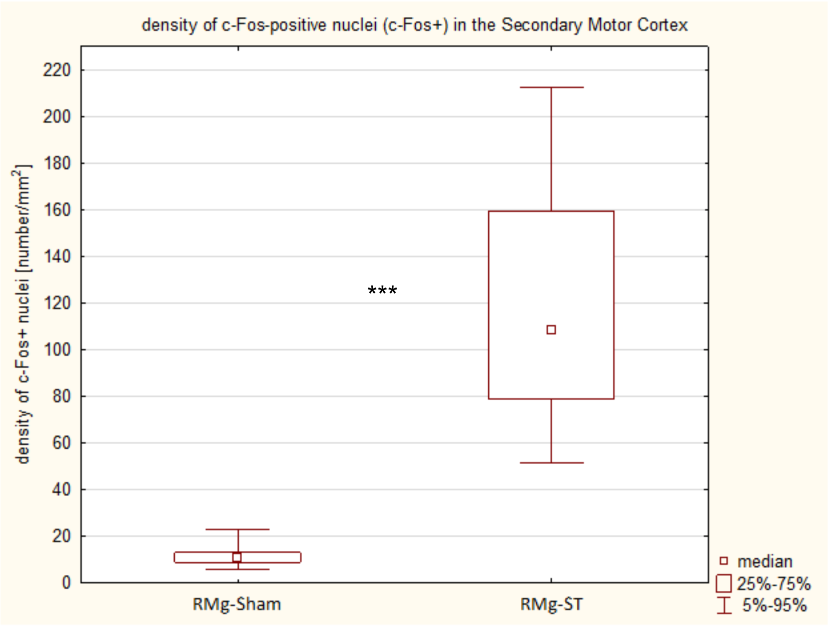

Figure S19. Comparison of the density of c-Fos-positive nuclei (c-Fos+) in the secondary motor cortex following electrical stimulation (RMg-ST) or a naïve proce-dure in control rats (RMg-Sham).
The Mann-Whitney U test indicates a significant difference between groups (*** $p < 0.001$).

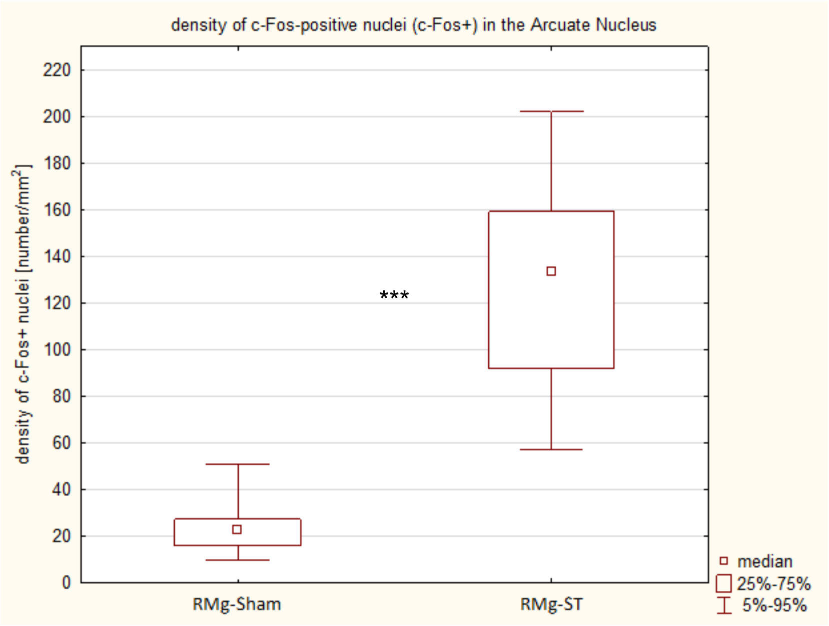

Figure S20. Comparison of the density of c-Fos-positive nuclei (c-Fos+) in the arcuate nucleus of the hypothalamus following RMg electrical stimulation (RMg-ST) or a naïve procedure in control rats (RMg-Sham).
The Mann-Whitney U test indicates a significant difference between groups (*** $p < 0.001$).

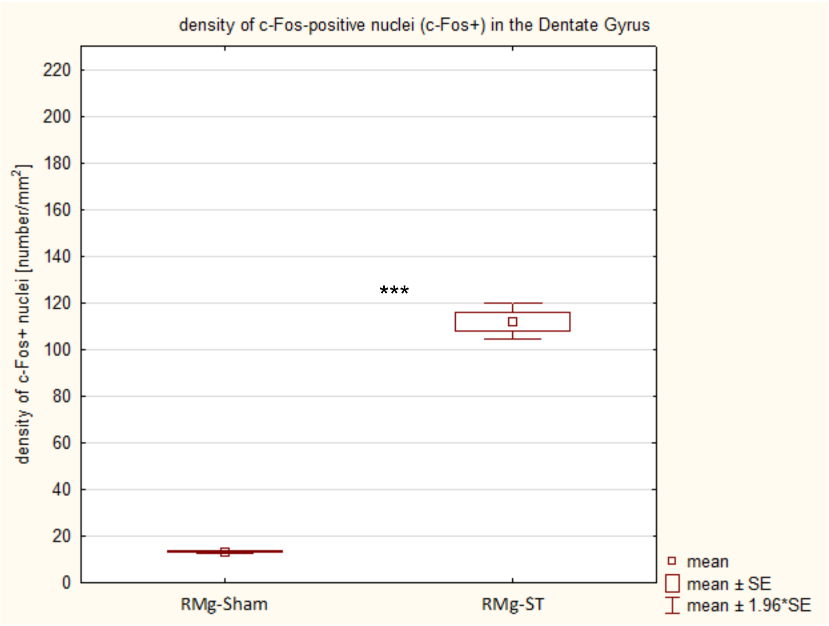

Figure S21. Comparison of the density of c-Fos-positive nuclei (c-Fos+) in the dentate gyrus of the hippocampus (DG) and the raphe magnus (RMg) in rats following RMg electrical stimulation (RMg-ST) or a naïve procedure in control rats (RMg-Sham).
The t-test indicates a significant difference between groups (*** $p < 0.001$).

Table S42. Results of the two-way ANOVA, including univariate tests of significance, effect sizes, and statistical power for the density of c-Fos–positive nuclei (c-Fos+) in selected brain structures following RMg electrical stimulation (RMg-ST) or a naïve procedure in control rats (RMg-Sham).

Effects	SS	degrees of freedom	MS	F	p
Intercept	5.8E+06	1	5.8E+06	3811.652	0.000
group	3.4E+06	1	3.4E+06	2222.397	0.000
structure	3.7E+05	3	1.2E+05	81.622	0.000
group × structure	2.1E+05	3	7.1E+04	46.684	0.000
Error	1.6E+06	1050	1.5E+03		

Table S43. Results of Levene’s test for the homogeneity of variance for the group × structure interaction in the density of c-Fos positive nuclei (c-Fos+) in selected brain structures following RMg electrical stimulation (RMg-ST) or naïve procedure in control rats (RMg-Sham).

Effects	SS	MS	F	p
Intercept	4.9E+04	5.2E+02	95.272	0.000

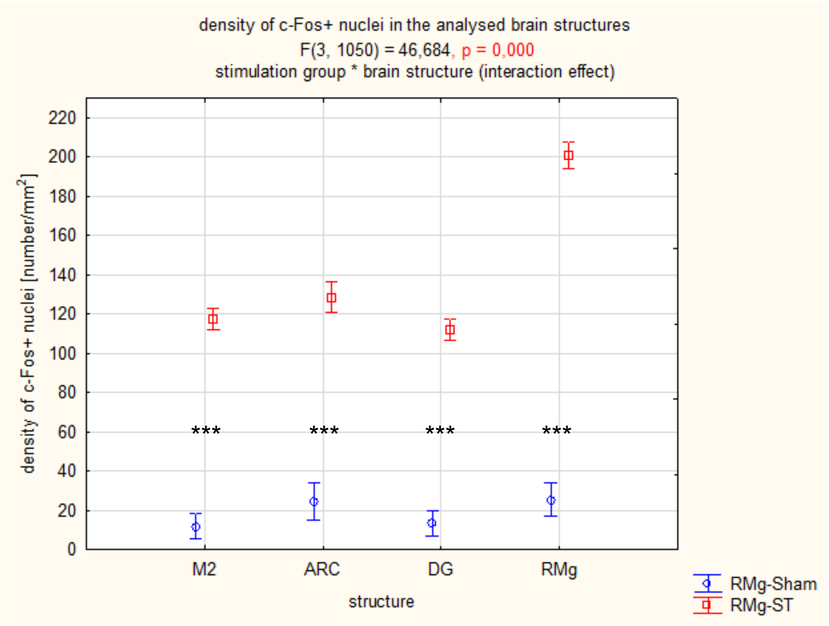

Figure S22. Comparison of the density of c-Fos-positive nuclei (c-Fos+) in selected brain structures: secondary motor cortex (M2), arcuate nucleus of the hypothalamus (ARC), dentate gyrus of the hippocamus (DG) and raphe magnus (RMg) in rats following RMg electrical stimulation (RMg-ST) or a naïve procedure in control (RMg-Sham) animals.

A two-way ANOVA confirms a significant (*** $p < 0.001$) interaction between stimulation group and brain structure ($F_{(3, 1050)} = 46.684$, $p = 0.000$)

Table S44. Games–Howell post-hoc analysis of the group × structure interaction for the density of c-Fos–positive (c-Fos+) nuclei in selected brain structures follow-ing RMg electrical stimulation (RMg-ST) or a naïve procedure in control (RMg-Sham) rats.

group	structure	{1}<br>11.69	{2}<br>24.38	{3}<br>13.42	{4}<br>25.42	{5}<br>117.31	{6}<br>125.58	{7}<br>112.10	{8}<br>200.82
RMg-Sham	M2		0.56	0.98	0.32	0.00	0.00	0.00	0.00
RMg-Sham	ARC	0.56		0.73	0.98	0.00	0.00	0.00	0.00
RMg-Sham	DG	0.98	0.73		0.50	0.00	0.00	0.00	0.00
RMg-Sham	RMg	0.32	0.98	0.50		0.00	0.00	0.00	0.00
RMg-ST	M2	0.00	0.00	0.00	0.00		0.44	0.88	0.00
RMg-ST	ARC	0.00	0.00	0.00	0.00	0.44		0.05	0.00
RMg-ST	DG	0.00	0.00	0.00	0.00	0.88	0.05		0.00
RMg-ST	RMg	0.00	0.00	0.00	0.00	0.00	0.00	0.00	

Table S45. Density of 5-HT-positive (5-HT+) cells in selected brain structures following RMg electrical stimulation (RMg-ST) or a naïve procedure in control rats (RMg-Sham).

[n – number of individuals per group, K-S states from Kolmogorov-Smirnov normality test (L – Liliefors significant level); SD states from the standard deviation; SE states from the standard error; IQR states from the interquartile range; t-test states from Student's t-test; U states from Mann-Whitney U test]

brain structure	group	n	mean	median	K-S	SD	SE	IQR	t-test	U
M2	RMg-Sham	136	15.8	17	D = 0.090<br>p > 0.20	6.55	0.56	8.4	**t = -37.341<br>p = 0.000**	**Z = -15.653<br>p = 0.000**
					L < 0.01				df 340	U = 0.0
	RMg-ST	206	111.8	106	D = 0.090<br>p < 0.10	29.49	2.05	37.2		
					L < 0.01					
ARC	RMg-Sham	67	27.1	28	D = 0.059<br>p > 0.20	8.89	1.09	11.4	**t = -21.978<br>p = 0.000**	**Z = -10.959<br>p = 0.000**
					L > 0.20				df 166	U = 0.0
	RMg-ST	101	148.8	146	D = 0.087<br>p > 0.20	44.71	4.45	44.0		
					L < 0.10					
DG	RMg-Sham	137	27.9	25	D = 0.118<br>p < 0.05	13.05	1.11	14.0	**t = -15.120<br>p = 0.000**	**Z = -15.683<br>p = 0.000**
					L < 0.05				df 343	U = 125.0
	RMg-ST	208	69.3	65	D = 0.123<br>p < 0.01	30.25	2.10	28.1		
					L < 0.01					

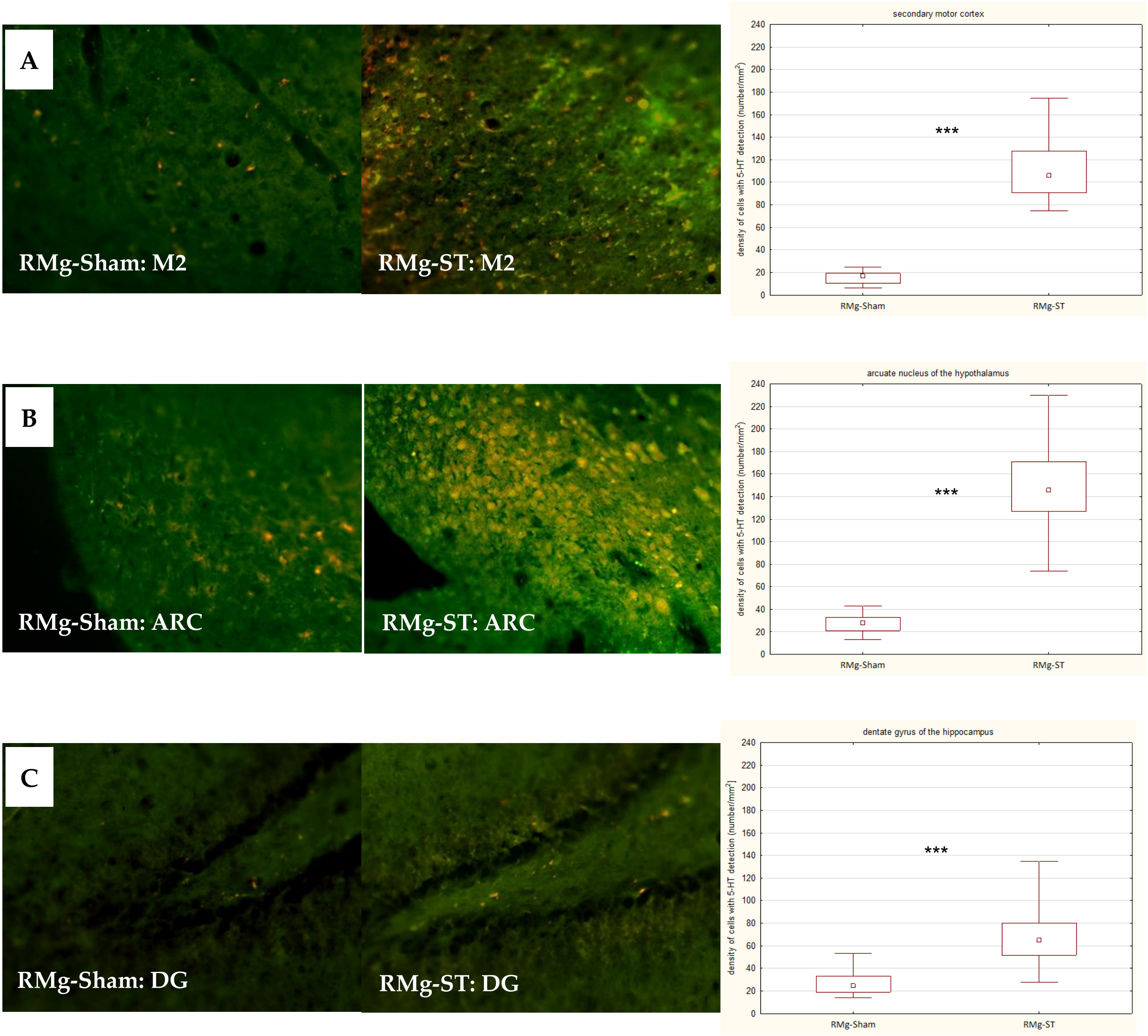

Figure S23. Micrographs of double staining with the density of 5-HT detection (green signal as Alexa Fluor 488) and with the density of c-Fos–positive (c-Fos+) nuclei (red signal as Alexa Fluor 546) of the secondary motor cortex (M2) (A), the arcuate nucleus of the hypothalamus (ARC) (B) and the dentate gyrus of the hippocampus (DG) (C) in the representative rats from control (RMg-Sham) and the stimulation (RMg-ST) groups. (PrimoStar fluorescence microscope from Carl Zeiss MicroImaging GmbH, Germany; image resolution 1024 × 1024 pixels; Axio Vision Rel 4.8 computer program from Carl Zeiss Imaging System; magnification 20 × 10). The box-and-whisker plot presents the density of cells with 5-HT detection [number/mm^2]. Boxes represents the interquartile range (25th–75th percentile), the horizontal line within each box indicates the median, and the whiskers denote the 5th-95th percentile range. Median values are marked with open squares. *** $p < 0.001$ indicates a significant difference between the RMg-ST and the RMg-Sham groups.
